# Efficacy of digital interventions for patients with post-stroke aphasia: a systematic review and network meta-analysis

**DOI:** 10.3389/fneur.2026.1936067

**Published:** 2026-10-12

**Authors:** Junjie Shi, Yue Sun, Jingyi Zhang, Zeyu Yang, Jia Hu, Liang Zhi, Qingfang Zhang, Yulong Wang, Jianjun Long

**Affiliations:** 1 School of Rehabilitation, Shandong University of Traditional Chinese Medicine, Jinan, Shandong, China; 2 Department of Rehabilitation, The Eighth Affiliated Hospital of Southern Medical University, Foshan, Guangdong, China; 3 Department of Rehabilitation, Shenzhen Second People’s Hospital, Dapeng Hospital, Shenzhen, Guangdong, China; 4 Department of Rehabilitation, Shenzhen Second People’s Hospital, Shenzhen, Guangdong, China; 5 Department of Rehabilitation, Shenzhen University of Advanced Technology General Hospital, Shenzhen, Guangdong, China

**Keywords:** aphasia, computer-assisted therapy, network meta-analysis, stroke, telerehabilitation, virtual reality

## Abstract

**Background:**

Digital interventions show potential for improving language function in post-stroke aphasia (PSA), but contradictory evidence leaves the optimal strategy unclear.

**Objective:**

This study aims to evaluate the effects of different digital interventions on language function and quality of life in patients with PSA, and to provide evidence-based support for clinical rehabilitation decisions.

**Methods:**

A systematic search across PubMed, Web of Science, Cochrane Library, Embase, Scopus, and China National Knowledge Infrastructure (CNKI) (inception to May 2026) identified randomized controlled trials (RCTs) evaluating digital interventions (e.g., virtual reality [VR], computer-assisted language therapy [CALT], telerehabilitation [TR]) for adults with PSA. Eligible studies reported Western Aphasia Battery or Stroke and Aphasia Quality of Life Scale-39 (SAQOL-39) outcomes. Data were extracted independently. Risk of bias was assessed using the Cochrane Risk of Bias 1 (RoB 1) tool. Network meta-analysis was performed using R 4.5.2, incorporating a consistency model, a random-effects model, and I^2^statistics to assess heterogeneity. Sources of heterogeneity were analyzed through sensitivity analyses, subgroup analyses, and meta-regression. Intervention efficacy was ranked using Surface Under the Cumulative Ranking Curve (SUCRA). Robustness was validated via the Egger test and sensitivity analyses. Evidence quality was assessed using the GRADE framework.

**Results:**

Twenty-five RCTs involving 1,069 participants were included. Compared to conventional therapy (C), VR showed significant superiority in aphasia quotient (mean difference [MD] = 10.77, 95% CI 9.48 to 12.06; SUCRA = 89.57%), spontaneous speech (MD = 2.35, 95% CI 1.71 to 2.99), auditory comprehension (MD = 1.47, 95% CI 0.81 to 2.13), and repetition (MD = 1.98, 95% CI 1.19 to 2.76); Ranking was VR > CALT>TR > C. For naming, CALT ranked highest (MD = 1.41, 95% CI 0.79 to 2.03; SUCRA = 89.87%); Ranking was CALT>VR > C > TR. For SAQOL-39, VR showed the best effect (MD = 0.51, 95% CI 0.33 to 0.70; SUCRA = 96.40%). Ranking was VR > TR > C > CALT.

**Conclusion:**

Digital interventions effectively improve language function in PSA. VR demonstrated potential advantages for aphasia quotient, speech, comprehension, repetition, and SAQOL-39, whereas CALT provides targeted advantages for naming impairments. Caution is warranted due to study heterogeneity, bias risks, and limited sample sizes. Future research should optimize intervention protocols, integrate neuromodulation or traditional therapeutic techniques, and explore cost-effective implementation strategies.

**Systematic review registration:**

https://www.crd.york.ac.uk/PROSPERO/view/CRD420261373199, Identifier CRD420261373199.

## Introduction

Post-stroke aphasia (PSA) is a common acquired language disorder that significantly impacts the functional recovery and quality of life of stroke survivors; up to one in three stroke patients is affected by it (1). Aphasia deficits typically span multiple linguistic dimensions, including impairments in verbal expression, auditory comprehension, reading and writing abilities (2). These impairments not only undermine patients’ ability to communicate linguistically but also lead to severe social isolation and psychological comorbidities such as depression and anxiety (3, 4). Crucially, without effective intervention, patients with PSA often experience severe disruptions to family life and social participation (5), highlighting the urgent need for effective rehabilitation strategies. In recent years, whilst traditional speech and language rehabilitation therapies (e.g., face-to-face exercises and augmentative and alternative communication tools) have been widely used in the clinical management of PSA, they are constrained by the uneven distribution of healthcare resources, high treatment costs and a reduction in treatment intensity following discharge. Clinical practice urgently requires personalized interventions that are more accessible and efficient, and which can precisely address the specific characteristics of patients’ language impairments.

Given the limitations of traditional therapies, digital therapies such as computer-assisted language therapy (CALT), virtual reality (VR) and telerehabilitation (TR) offer new approaches to overcoming these challenges. CALT primarily relies on intelligent algorithms and adaptive learning systems to deliver high-frequency, personalized training whilst dynamically adjusting task difficulty (6), and has demonstrated significant potential in improving functional communication skills such as spontaneous speech, auditory comprehension and naming (7). As the core of CALT, adaptive learning systems dynamically adjust task difficulty based on real-time performance data, ensuring that the training difficulty is matched to the patient’s level and that tasks remain both challenging and achievable. VR utilizes immersive virtual environments to combine multimodal sensory stimulation with task-oriented training (8), creating training scenarios that closely resemble everyday life for patients, thereby enhancing their engagement in training and their ability to process language in contextual settings. By integrating multimodal stimuli (e.g., visual, auditory, and tactile inputs), VR creates a comprehensive human-computer interaction environment that promotes neuroplasticity (9). TR primarily utilizes video conferencing platforms (10) and mobile applications to deliver remote interventions via information and communication technology (11), enabling patients to receive continuous language training in their home environment. This overcomes constraints related to healthcare resources and geographical location, thereby significantly improving access to rehabilitation (12, 13). Furthermore, considering the potential clinical overlap among these interventions, it is essential to delineate them based on their primary therapeutic objectives. Clarifying their core active components helps establish the conceptual independence required for the subsequent network synthesis.

Previous studies have indicated that digital therapy can, to a certain degree, enhance the language function and communication abilities of patients with PSA. For instance, VR exhibits potential advantages in functional communication and language engagement (14, 15), whereas CALT has shown promising results in naming tasks and the reinforcement of language practice (16, 17). Nevertheless, the majority of current systematic reviews and meta-analyses primarily focus on comparing the efficacy of individual digital therapies against conventional treatments (18–20), leaving a gap in systematic evidence regarding the efficacy of various digital interventions across distinct language domains. Furthermore, notable heterogeneity exists among studies in terms of intervention dosage, duration, and outcome measures, which contributes to inconsistent research findings. In clinical practice, clinicians may need to select among different digital interventions based on patients’ specific language impairments and rehabilitation needs. However, the lack of direct comparisons limits the evidence regarding which interventions may offer greater benefits for specific language domains, highlighting the need for comparative evaluation of their effects.

Network meta-analysis (NMA) can synthesize both direct and indirect evidence, enabling comparison and ranking of the effects of multiple treatments to identify the relative effects of different digital interventions across various language domains. Therefore, this study aims to compare the efficacy of CALT, VR, and TR in patients with PSA through a systematic review and NMA, and evaluate their effects on the Aphasia Quotient (AQ) from the Western Aphasia Battery (WAB) scale, as well as on spontaneous speech, auditory comprehension, repetition, and naming, in addition to the Stroke and Aphasia Quality of Life Scale-39 (SAQOL-39). Specifically, we hypothesized that digital interventions would demonstrate greater improvements than conventional therapy, and that different digital interventions may show outcome-specific advantages. The ultimate goal is to provide evidence-based support for the clinical application of appropriate digital rehabilitation strategies for PSA.

## Methods

### Protocol and registration

This study constitutes a systematic review and network meta-analysis, with its protocol duly registered in PROSPERO (CRD420261373199) and adhering rigorously to the PRISMA 2020 (Preferred Reporting Items for Systematic Reviews and Meta-Analyses) guidelines. Given that all data incorporated in this study are sourced exclusively from published randomized controlled trials, ethical approval is deemed unnecessary.

### Eligibility criteria

Studies were selected based on predefined eligibility criteria established before the study search. The criteria were developed to identify randomized controlled trials evaluating the effects of digital interventions for post-stroke aphasia. The specific inclusion and exclusion criteria are described below.

### Inclusion criteria

The selection and inclusion of studies in this research adhered to the PICOS (Population, Intervention, Comparison, Outcome, and Study Design) principles (21), with specifics outlined in Table 1.

**Table 1:** Inclusion criteria.

PICOS elements	Inclusion criteria
Population	Adult patients (aged >18 years) clinically diagnosed with stroke and presenting with post-stroke aphasia
Intervention	The core intervention consisted of digital therapeutic interventions, including computer-assisted language therapy, virtual reality, and telerehabilitation
Comparator	The control group received either conventional speech-language therapy, other digital interventions, or usual care/blank control.
Outcomes	Original studies must report at least one outcome of interest, such as the Western Aphasia Battery (WAB) Aphasia Quotient (AQ) or its subscale scores (Spontaneous Speech, Auditory Comprehension, Repetition, Naming), and the Stroke and Aphasia Quality of Life Scale (SAQOL−39).
Study Design	Randomized controlled trial

### Exclusion criteria

The exclusion criteria pertaining to participants, interventions, comparators, outcomes, and study design are presented in Table 2. To ensure the independence of interventions and prevent confounding, trials evaluating inseparable co-administered digital interventions (e.g., CALT combined with TR) where the primary active component could not be isolated were explicitly excluded. Furthermore, to eliminate potential treatment contamination, studies were excluded if the conventional therapy incorporated computer-assisted or other digital elements.

**Table 2:** Exclusion criteria.

PICOS elements	Exclusion criteria
Population	Patients with aphasia not caused by stroke (e.g., cranial brain injury, neurodegenerative diseases) Patients with severe cognitive impairment, audiovisual impairment, or severe mental illness Minors
Intervention	Non-digital interventions (e.g., pharmacological interventions, acupuncture) Digital technology used solely for augmentative and alternative communication (AAC) Co-administered digital interventions (e.g. CALT + TR) where the primary active component cannot be isolated
Comparator	Control group with unconventional therapies (e.g., pharmacological or surgical interventions) Studies in which the control group received additional therapies (e.g., pharmacological treatment) not administered to the intervention group Conventional therapy control group that explicitly incorporate computer-assisted tools, virtual reality, or any other digital elements.
Outcomes	Endpoints are reported in nonquantitative forms (e.g., qualitative descriptions) Missing baseline or endpoint data precluding calculation of effect sizes via formulas
Study design	Reviews, conference abstracts, case reports, and non − randomized controlled trial designs

### Information sources

We performed a systematic search of databases including PubMed, Web of Science, Cochrane Library, Embase, Scopus, and China National Knowledge Infrastructure (CNKI). Because this study is an original systematic review and network meta-analysis rather than an update of a previous review, no earlier pool of included studies was carried forward (recorded as *n* = 0 in the ‘Previous studies’ panel of the PRISMA search flow diagram). The comprehensive literature search was completed in May 2026, and the subsequent study screening process was completed in June 2026. Furthermore, supplementary searches were also conducted via Google Scholar and manual citation searching.

### Search strategy

To ensure the search’s rigor and the replicability of the methodology, two independent researchers formulated and implemented a detailed search strategy. Considering the variations in syntax, indexing systems, and terminology across different databases, we tailored each search query accordingly. The search strategy integrated both MeSH (Medical Subject Headings) and EmTree terms (e.g., Stroke, Aphasia, VR, and Telemedicine), along with free-text terms (e.g., PSA, CALT, VR, and Telerehabilitation). For the CNKI database, the search strategy was developed in Chinese and adapted to the database’s indexing and retrieval framework. The Chinese search strategy and its English translation are provided in Supplementary File 1. The application of Boolean operators and adjustments to advanced search syntax strictly adhered to the specifications of each database, without imposing any restrictions on publication year (i.e., anytime to present) or language. The complete search queries for each database are detailed in Supplementary File 1. Supplementary searches involved screening Google Scholar and reference lists of candidate RCTs and related systematic reviews for additional PICO-eligible records.

### Study selection

In this analysis, all retrieved records were imported into EndNote X9 (Clarivate Analytics) for literature management and duplicate removal. Records identified via supplementary searches were cross-referenced against primary database results at the identification stage. Overlapping records were deduplicated and retained in the primary database pool to avoid double-counting (a detailed audit trail mapping these supplementary records to their citation sources and database identifiers is provided in Supplementary File 2). The included literature was screened according to the pre-established inclusion and exclusion criteria. Before the formal screening process, the two researchers discussed the eligibility criteria and screening procedures to ensure a consistent understanding of the selection criteria. Subsequently, the tasks of study screening, quality evaluation, and data extraction were all independently carried out by two researchers. Any discrepancies arising during the screening process were addressed through discussion or consultation with a third researcher.

### Data extraction and data items

Based on predefined inclusion and exclusion criteria, two researchers independently extracted and reviewed data from eligible studies; any disagreements were resolved through consultation with a third researcher. Relevant data were systematically collected, including study titles, first authors, publication years, participant characteristics, sample sizes, duration of intervention, intervention details, outcome measures, and quality assessments. When relevant data were unclear or unavailable in the published articles, the corresponding authors were contacted for further clarification. In this study, one corresponding author was contacted and provided the requested information, which was subsequently incorporated into the data extraction process.

### Risk of bias assessment

This study used the Cochrane Risk of Bias 1 (RoB 1) tool to assess the methodological quality of the included studies. We evaluated seven core dimensions of bias: bias arising from random sequence generation; bias arising from allocation concealment; bias arising from blinding of participants and personnel; bias arising from blinding of outcome assessment; bias arising from incomplete outcome data; bias arising from selective reporting; and bias arising from other sources. Studies were classified as (1) low risk: all domains were rated as “low risk”; (2) unclear: at least one domain was rated as having “unclear risk,” but no domain was rated as “high risk”; and (3) high risk: at least one domain was rated as “high risk.” Importantly, all studies meeting the eligibility criteria were included regardless of their risk of bias scores. The risk assessment was utilized strictly to appraise the methodological quality of the evidence and to inform data interpretation, rather than serving as an exclusion criterion.

### Synthesis methods and statistical analysis

This analysis used the “netmeta” package in R version 4.5.2 under a frequentist framework. Since the WAB and the SAQOL-39 used in this study are both continuous variables, clinical effect sizes were quantified using mean differences (MDs) and their 95% confidence intervals. Heterogeneity among studies was quantified using the I^2^ statistic, with I^2^ ≤ 50% and I^2^ > 50% defined as low and high heterogeneity, respectively. Given the expected clinical and methodological heterogeneity among the included studies in terms of baseline population characteristics, intervention protocols, and intervention durations, all network meta-analyses were conducted using a random-effects model. When substantial heterogeneity was observed, pre-specified subgroup analyses and meta-regressions were conducted based on stroke phase, age, and cumulative intervention time.

To ensure node independence and conceptual clarity, interventions were categorized by their primary active component. Given that CALT and VR represent therapeutic technologies while TR functions as a delivery method, studies with overlapping features were assigned based on their main active component. Additionally, to prevent cross-arm contamination, conventional therapy (C) controls were strictly restricted to traditional, non-digital, face-to-face intervention. For multi-arm studies, intervention arms were assigned to the corresponding network nodes based on their intervention modalities. For example, Zhou et al. (22) was a four-arm study that included separate CALT and TR comparisons, each with a distinct conventional rehabilitation control arm. The study was treated as a single multi-arm study rather than as two independent studies. Additionally, the transitivity assumption was considered when incorporating different intervention comparisons from the same study into the network, particularly with respect to the comparability of relevant clinical and methodological characteristics.

The validity of network meta-analysis (NMA) relies on the assumptions of consistency between direct and indirect evidence and transitivity across the entire evidence network. For networks containing closed loops, global inconsistency is generally assessed using the Design-by-treatment interaction model to evaluate potential discrepancies between different sources of evidence. A *p*-value >0.05 indicates no significant inconsistency, supporting the use of a consistency model; otherwise, an inconsistency model should be considered, with the node-splitting method applied to further assess local inconsistency. In the present study, the constructed evidence network did not contain any closed loops, indicating the absence of direct comparisons between interventions and precluding statistical evaluation of inconsistency. Therefore, the consistency model was adopted for effect estimation and quantitative synthesis. Forest plots are used to compare the effect sizes of interventions versus conventional therapy (C), and network diagrams are used to visualize the connections between different treatment measures. Intervention effects are ranked by SUCRA scores, with higher percentages indicating superior efficacy, and pairwise comparison results between interventions are presented in a league table. We assessed the presence of publication bias using funnel plot symmetry combined with the Egger test. Additionally, to evaluate the robustness of the network results, we conducted sensitivity analyses by sequentially excluding individual studies using the stepwise exclusion method. If overall heterogeneity decreased significantly after excluding a particular study, this indicated that the study was likely the primary source of heterogeneity.

### Certainty assessment

This analysis uses the GRADE framework to systematically assess the quality of evidence for each outcome. Based on factors such as the risk of bias in the original studies, heterogeneity and non-transferability, inconsistency, imprecision, or publication bias, the level of evidence is downgraded stepwise from high to low. Ultimately, the quality of evidence for each study is objectively assessed as one of four levels: high, moderate, low, or very low. For details, see Supplementary File 3.

## Results

### Literature search results

A total of 3,795 articles were retrieved from six databases: PubMed (*n* = 253), Cochrane Library (*n* = 2,072), Web of Science (*n* = 655), Scopus (*n* = 90), Embase (*n* = 250), and CNKI (*n* = 475). Additionally, supplementary searches identified 12 records, all overlapped with primary database hits and were deduplicated at the identification stage (Supplementary File 2). After removing 1,668 duplicates, the remaining 2,127 articles were screened based on their titles and abstracts. Of these, 1,991 were excluded for irrelevance. The full texts of 136 articles were reviewed, and 111 were excluded for failing to meet the inclusion criteria. Ultimately, 25 RCTs were included. Among these, 11 studies had also been covered in previously published independent systematic reviews. The detailed search flow is illustrated in Figure 1.

**Figure 1:**
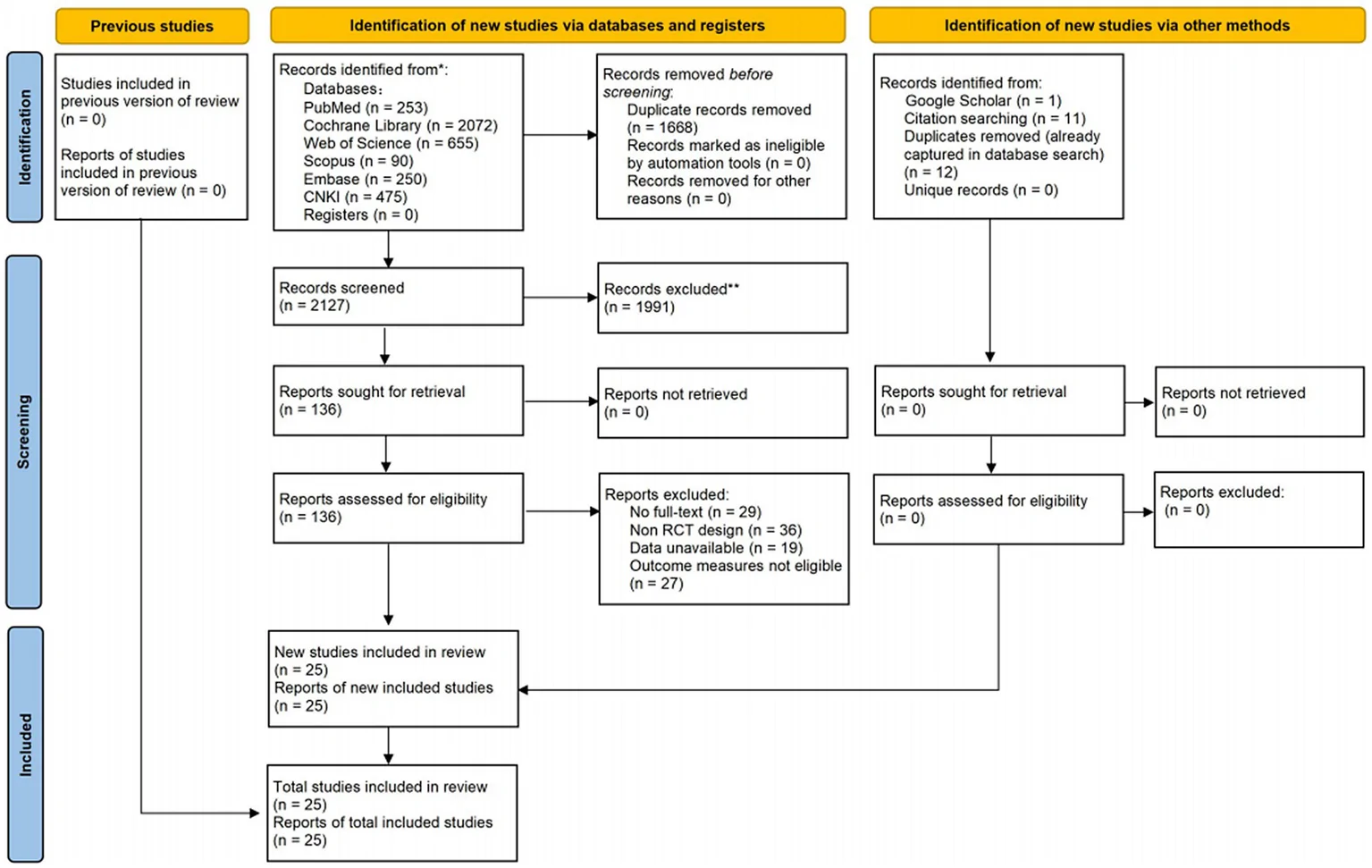
PRISMA search flow diagram of the study selection process for the systematic review and network meta-analysis of digital interventions for post-stroke aphasia. The flow diagram is structured into three parallel panels in accordance with PRISMA 2020 standards. Because this study represents an original (*de novo*) systematic review rather than an update of a previously published review, no prior studies were carried forward (Previous studies: *n* = 0). Primary electronic searches across six databases yielded 3,795 records. Supplementary searches via Google Scholar and manual citation searching identified 12 candidate records; however, as all 12 records completely overlapped with primary database hits, they were deduplicated at the identification stage, resulting in 0 unique records requiring separate screening through the “other methods” pathway” (detailed in Supplementary File 2).

### Study characteristics

This analysis included a total of 25 studies involving 1,069 patients with PSA. The studies involved three different digital interventions: CALT, VR, and TR. According to our predefined classification criteria, each intervention node was explicitly delineated, ensuring no confounding overlap that could compromise the data synthesis. Twelve studies involved CALT, nine involved VR, and five involved TR. The baseline characteristics of the included studies are shown in Table 3. The reported outcome data for AQ, spontaneous speech, auditory comprehension, repetition, naming, and SAQOL-39 are summarized in Supplementary File 4.

**Table 3:** Characteristics of the included randomized controlled trials evaluating digital interventions for post-stroke aphasia (PSA) in adults.

Study (year) and group	Country/Region	Disease duration, mean (SD)	Age (years), mean (SD)	Intervention	Intervention system or intervention modality	Sample size	Time	Outcome indicators	Measuring instrument
Kesav et al. (23) (2017)	India								
TG^a^		31.2 (31) days	56.27 (11.62)	CALT^b^	MOZHI	11	4 weeks	①	WAB^c^
CG^d^		25.3 (30) days	48.67 (11.83)	C^e^	Speech and language therapy	9	4 weeks	①	WAB
Alam et al. (24) (2021)	India								
TG		9.8 (2.38) months	50.8 (3.19)	CALT	Jellow app	5	6 months	①②③④⑤⑥	WAB and SAQOL-39^f^
CG		10.6 (2.07) months	51.6 (3.05)	C	Schuell stimulation	5	6 months	①②③④⑤⑥	WAB and SAQOL-39
Liu et al. (25) (2022)	China								
TG		28.3 (14.5) days	51.53 (11.09)	CALT	Wispirit Ltd. computerized training module	33	4 weeks	①②③④⑤	WAB
CG		30.2 (18) days	54.33 (12.76)	C	Speech and language therapy	35	4 weeks	①②③④⑤	WAB
Cherney (26) (2010)	USA								
TG		66.7 (71.5) months	56.6 (9.2)	CALT	Computer ORLA	11	8 weeks	①	WAB
CG		41.3 (45.7)months	61.1 (14.8)	C	SLP-ORLA	14	8 weeks	①	WAB
Gan et al. (12) (2025)	China								
TG		3.49 (1.65) months	49.57 (10.91)	TR^g^	TR-ORLA	14	3 months	①⑥	WAB and SAQOL-39
CG		3.44 (1.78) months	48.79 (11.09)	C	Offline-ORLA	14	3 months	①⑥	WAB and SAQOL-39
Marshall et al. (27) (2020)	UK								
TG		48 (41.11) months	51 (8.15)	VR^h^	EVA Park	16	6 months	①⑥	WAB and SAQOL-39
CG		26.5 (49.81) months	65 (14.63)	C	Routine nursing	18	6 months	①⑥	WAB and SAQOL-39
Zhou et al. (22) (2018)	China								
TG		34.8 (20.65) days	58.60 (11.44)	CALT	computerized speech−language and cognitive training	10	2 weeks	①②③④⑤	WAB
CG		29.9 (19.73) days	56.10 (17.29)	C	Routine rehabilitation training	10	2 weeks	①②③④⑤	WAB
Zhou et al. (22) (2018)	China								
TG		32 (17.06) days	59.80 (11.26)	TR	Wispirit Inc. (66nao.com)	10	2 weeks	①②③④⑤	WAB
CG		32.8 (19.89) days	56.50 (14.34)	C	Family topics communication	10	2 weeks	①②③④⑤	WAB
Braley et al. (28) (2021)	USA								
TG		53 (56) months	58.9 (10)	TR	Constant Therapy-Resarch	17	10 weeks	①②③④⑤⑥	WAB and SAQOL-39
CG		38.1 (32) months	64.2 (9.9)	C	Standard of care (SOC) speech- language pathology workbook	15	10 weeks	①②③④⑤⑥	WAB and SAQOL-39
Li et al. (29) (2024)	China								
TG		3.54 (0.79) months	63.71 (7.91)	VR	Immersive virtual reality speech rehabilitation system	45	12 weeks	①②③④⑤⑥	WAB and SAQOL-39
CG		3.26 (0.87) months	62.58 (7.54)	C	Routine rehabilitation training	45	12 weeks	①②③④⑤⑥	WAB and SAQOL-39
Chen et al. (30) (2025)	China								
TG		3.93 (1.07) months	50.91 (5.92)	CALT	Ai-based intensive speech and language therapy	11	2 weeks	①②③④⑤⑥	WAB and SAQOL-39
CG		3.86 (0.89) months	58.45 (10.49)	C	Routine rehabilitation training	11	2 weeks	①②③④⑤⑥	WAB and SAQOL-39
Shao et al. (31) (2025)	China								
TG		35.68 (5.90) days	59.38 (6.12)	VR	Mirror neuron action observation and imitation therapy based on virtual reality	53	4 weeks	①②③④⑤	WAB
CG		35.17 (5.58) days	58.93 (6.57)	C	Routine rehabilitation training	53	4 weeks	①②③④⑤	WAB
Chen et al. (32) (2025)	China								
TG		NOR^i^	NOR	CALT	Video teaching	30	30 days	②③④	WAB
CG		NOR	NOR	C	Routine rehabilitation training	30	30 days	②③④	WAB
Ye et al. (33) (2021)	China								
TG		13.6 (2.09) days	65.11 (6.92)	VR	VR-PHT-M1	30	8 weeks	①②③④⑤	WAB
CG		14.1 (2.01) days	64.98 (7.13)	C	Speech and language therapy	30	8 weeks	①②③④⑤	WAB
Qian et al. (34) (2023)	China								
TG		NOR	NOR	CALT	Computerized executive function training	30	4 weeks	①②③④⑤	WAB
CG		NOR	NOR	C	Routine rehabilitation training	30	4 weeks	①②③④⑤	WAB
Xiong et al. (35) (2021)	China								
TG		21 (11.11) days	53.75 (10.73)	CALT	Smart phone speech training	20	4 weeks	①②③④⑤	WAB
CG		27.5 (21.85) days	57.50 (10.84)	C	Routine rehabilitation training	20	4 weeks	①②③④⑤	WAB
Katz et al. (36) (1992)	USA								
TG		6.82 (5.47) years	59.53 (6.07)	CALT	Computer reading treatment software	15	6 months	①	WAB
CG		3.77 (1.38) years	65.64 (4.52)	C	Computer stimulation software	15	6 months	①	WAB
Katz et al. (37) (1997)	USA								
TG		6.2 (5.2) years	61.6 (10.0)	CALT	Computer reading treatment software	21	26 weeks	①②③④⑤	WAB
CG		5.4 (4.6) years	66.4 (6.0)	C	Computer stimulation software	19	26 weeks	①②③④⑤	WAB
Peñaloza et al. (38) (2021)	USA								
TG		52.37 (79.28) months	59.23 (18.71)	TR	ZOOM	8	10 weeks	①	WAB
CG		86.62 (127.89) months	54.63 (16.73)	C	Routine rehabilitation training	8	10 weeks	①	WAB
Fuentes et al. (39) (2024)	Spain								
TG		23.44 (42.4) months	68 (9.63)	CALT	DULCINEA	10	3 months	①⑥	WAB and SAQOL-39
CG		36.2 (49.9) months	64.5 (13.93)	C	Routine rehabilitation training	11	3 months	①⑥	WAB and SAQOL-39
Kurul et al. (40) (2026)	Turkey								
TG		NOR	74.02 (10.73)	VR	Meta Quest 2	46	8 weeks	⑥	SAQOL-39
CG		NOR	71.45 (12.65)	C	Routine rehabilitation training	46	8 weeks	⑥	SAQOL-39
Chen et al. (41) (2021)	China								
TG		NOR	54.6 (9.63)	VR	MNST—V1.0	15	2 weeks	①②③④⑤	WAB
CG		NOR	58.13 (11.11)	C	Routine rehabilitation training	15	2 weeks	①②③④⑤	WAB
You et al. (42) (2019)	China								
TG		69.48 (52.59) days	56.52 (12.29)	VR	Mirror neuron rehabilitation training system output device	21	4 weeks	②③④⑤	WAB
CG		70.90 (51.89) days	58.71 (12)	C	Routine rehabilitation training	21	4 weeks	②③④⑤	WAB
Xu et al. (43) (2020)	China								
TG		25.6 (8.74) days	61.62 (9.2)	VR	MNSTV1.0	20	4 weeks	①②③④⑤	WAB
CG		23.5 (9.91) days	58.25 (11.40)	C	Routine rehabilitation training	20	4 weeks	①②③④⑤	WAB
Tian et al. (44) (2017)	China								
TG		51.3 (14.4) days	59.3 (12.4)	VR	MNSTV1.0	15	4 weeks	①②③④⑤	WAB
CG		48.8 (17.6) days	62.1 (10.7)	C	Routine rehabilitation training	15	4 weeks	①②③④⑤	WAB
Meltzer et al. (45) (2018)	Canada								
TG		NOR	66.8 (11.2)	TR	WebEx	16	10 weeks	①②③④⑤	WAB
CG		NOR	62.9 (11.6)	C	Routine rehabilitation training	17	10 weeks	①②③④⑤	WAB

^a^TG, treatment group; ^b^CALT, computer-assisted language therapy; ^c^WAB, Western Aphasia Battery; ^d^CG, control group; ^e^C, conventional therapy (speech and language therapy, routine rehabilitation training, and nursing care); ^f^SAQOL-39, Stroke and Aphasia Quality of Life Scale-39; ^g^TR, telerehabilitation; ^h^VR, virtual reality; ^i^NOR, not otherwise reported. ①: aphasia quotient, ②: spontaneous speech, ③: auditory comprehension, ④: repetition, ⑤: naming, ⑥: SAQOL−39 score.

### Risk of bias

This analysis included a total of 25 studies (RCTs), of which 9 were rated as high risk, 3 as low risk, and the remaining 13 as unclear risk. The analysis revealed that most studies had shortcomings in randomization and allocation concealment, which may have introduced some degree of bias. Most studies had a low risk of bias regarding outcome measurement and selective reporting; however, the risk of other potential sources of bias could not be clearly assessed in most cases and was therefore rated as moderate. Detailed risk of bias assessments are shown in Figure 2 (12, 22–46).

**Figure 2:**
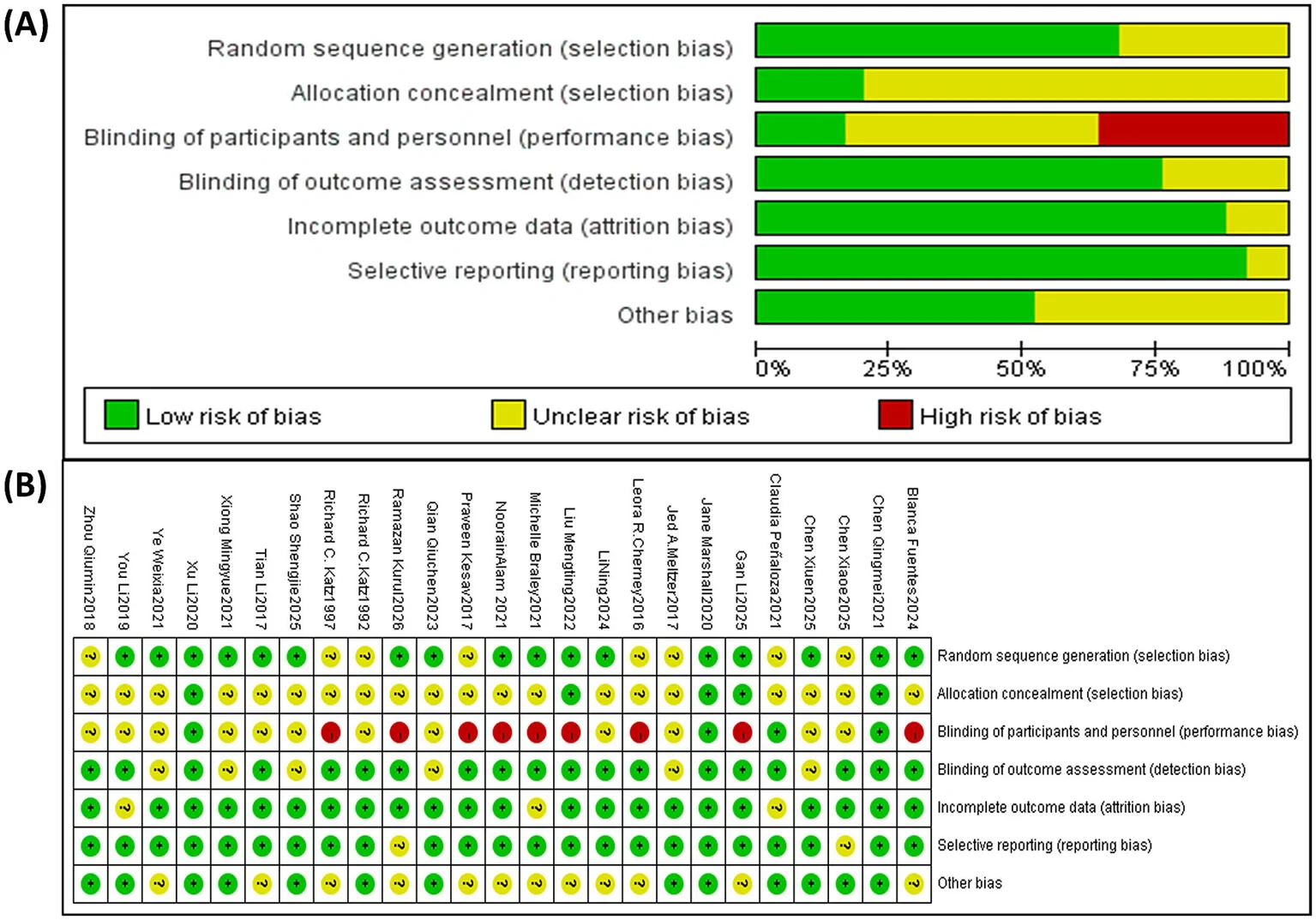
Risk of bias for the included trials. Risk of bias graph **(A)**. Risk of bias summary **(B)**.

### Network meta-analysis

#### Network evidence relationship

Network graphs were constructed using mean differences (MDs) with 95% confidence intervals (CIs), and the evidence network for each outcome is presented in Figure 3. As no closed loops were identified in any of the network plots, inconsistency testing was deemed unnecessary, and a consistency model was therefore adopted.

**Figure 3:**
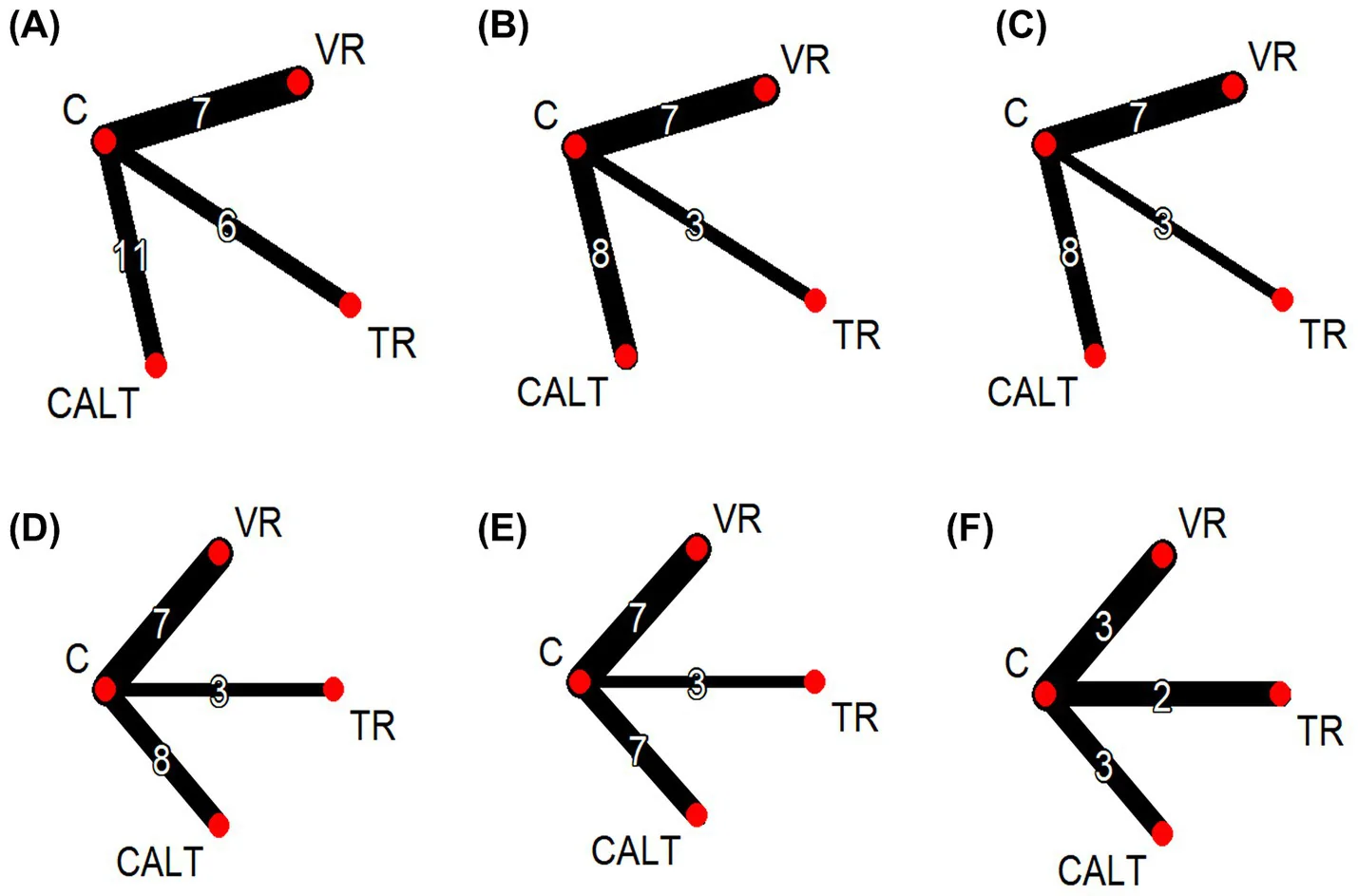
Network evidence diagram of included studies. C, conventional therapy (speech and language therapy, routine rehabilitation training, and nursing care); CALT, computer-assisted language therapy; VR, virtual Reality; TR, telerehabilitation. **(A)** Aphasia quotient; **(B)** spontaneous speech; **(C)** auditory comprehension; **(D)** repetition; **(E)** naming; **(F)** SAQOL-39.

#### AQ score

In the AQ assessment, VR showed the greatest improvement compared to C (MD = 10.77, 95% CI 9.48 to 12.06; SUCRA = 89.57%). CALT ranked second (MD = 10.01, 95% CI 7.25 to 12.77; SUCRA = 76.97%). TR did not show a significant difference compared to C (Figure 4A; Table 4).

**Figure 4:**
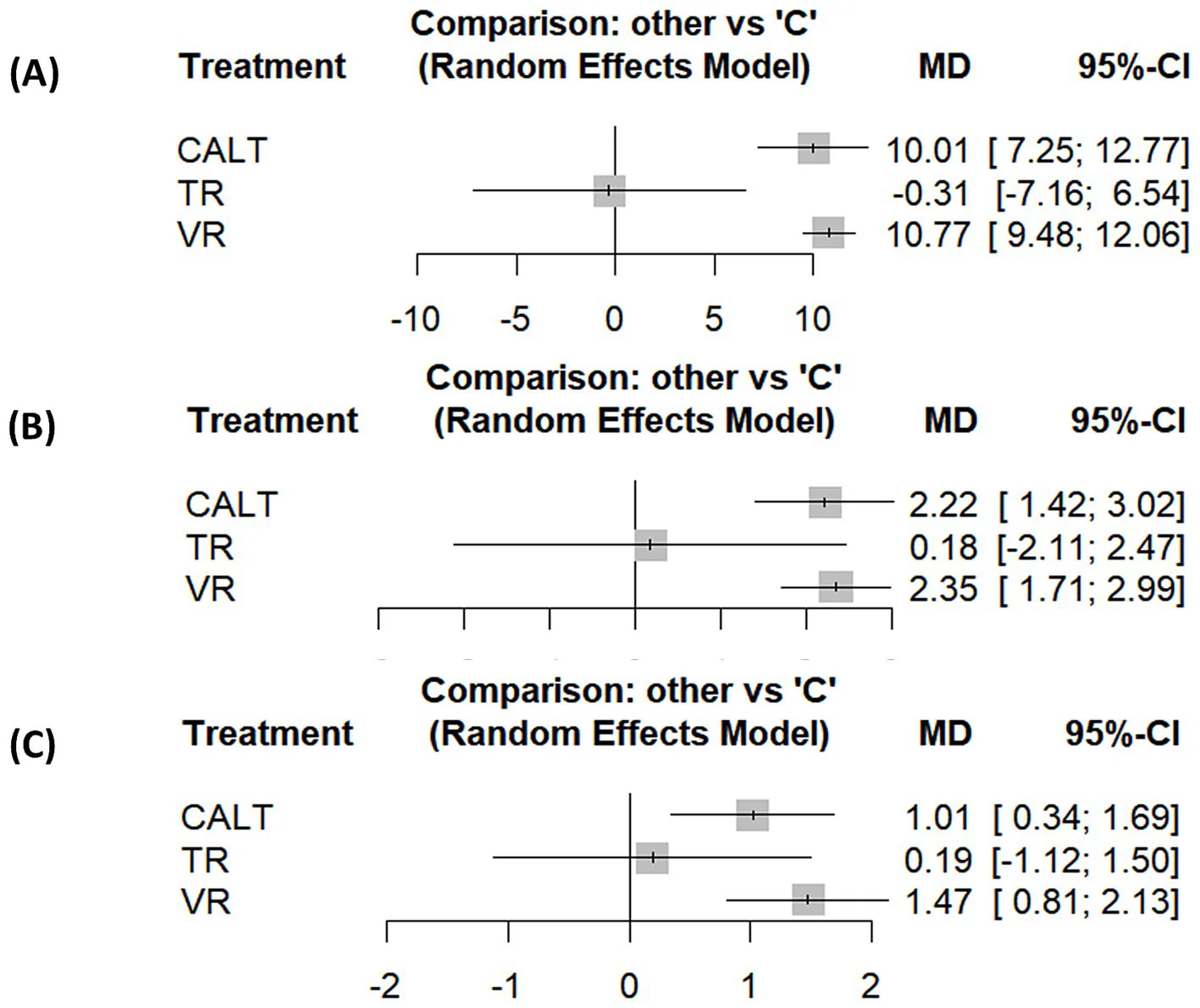
Forest plot for aphasia quotient **(A)**, spontaneous speech **(B)**, and auditory comprehension **(C)**. C, conventional therapy (speech and language therapy, routine rehabilitation training, and nursing care); CALT, computer-assisted language therapy; VR, virtual reality; TR, telerehabilitation; MD, mean difference.

**Table 4:** League table for the aphasia quotient (lower left) and spontaneous speech (upper right) showing the mean differences in scores and their 95% CIs among the interventions.

Treatment	Spontaneous speech			
AQ	C^a^	−0.18 (−2.47, 2.11)	−2.22 (−3.02, −1.42)	−2.35 (−2.99, −1.71)
	−0.31 (−7.16, 6.54)	TR^b^	−2.04 (−4.46, −0.19)	−2.17 (−4.55, −0.20)
	−10.01 (−12.77, −7.25)	−10.32 (−17.70, −2.94)	CALT^c^	−0.13 (−1.16, 0.89)
	−10.77 (−12.06, −9.48)	−11.08 (−18.05, −4.11)	−0.76 (−3.8, 2.28)	VR^d^

^a^C, conventional therapy (speech and language therapy, routine rehabilitation training, and nursing care); ^b^TR, telerehabilitation^c;^ CALT, computer−assisted language therapy; ^d^VR, virtual reality.

#### Spontaneous speech

For spontaneous speech, VR was ranked first compared to C (MD = 2.35, 95% CI 1.71 to 2.99; SUCRA = 85.45%). CALT ranked second (MD = 2.22, 95% CI 1.43 to 3.02; SUCRA = 78.34%). TR did not differ significantly from C (Figure 4B; Table 4).

#### Auditory comprehension

In the auditory comprehension assessment, VR showed a clear advantage over C and was ranked first (MD = 1.47, 95% CI 0.81 to 2.13; SUCRA = 92.80%). CALT was ranked second (MD = 1.01, 95% CI 0.34 to 1.69; SUCRA = 67.82%). There was no significant difference between TR and C (Figure 4C; Table 5).

**Table 5:** League table for the auditory comprehension (lower left) and repetition (upper right) showing the mean differences in scores and their 95% CIs among the interventions.

Treatment	Repetition			
Auditory Comprehension	C^a^	−0.16 (−1.73, 1.40)	−1.32 (−2.15, −0.49)	−1.98 (−2.76, −1.19)
	−0.19 (−1.50, 1.12)	TR^b^	−1.16 (−2.93, 0.61)	−1.81 (−3.57, −0.06)
	−1.01 (−1.69, −0.34)	−0.82 (−2.30, 0.65)	CALT^c^	−0.65 (−1.80, 0.49)
	−1.47 (−2.13, −0.81)	−1.28 (−2.74, −0.07)	−0.46 (−1.40, 0.49)	VR^d^

^a^C, conventional therapy (speech and language therapy, routine rehabilitation training, and nursing care); ^b^TR, telerehabilitation; ^c^CALT, computer-assisted language therapy; ^d^VR, virtual reality.

#### Repetition

For repetition, VR ranked first compared with C (MD = 1.98, 95% CI 1.19 to 2.76; SUCRA = 94.89%). CALT remained second (MD = 1.32, 95% CI 0.49 to 2.15; SUCRA = 67.70%). TR did not demonstrate a significant benefit. (Figure 5A; Table 5).

**Figure 5:**
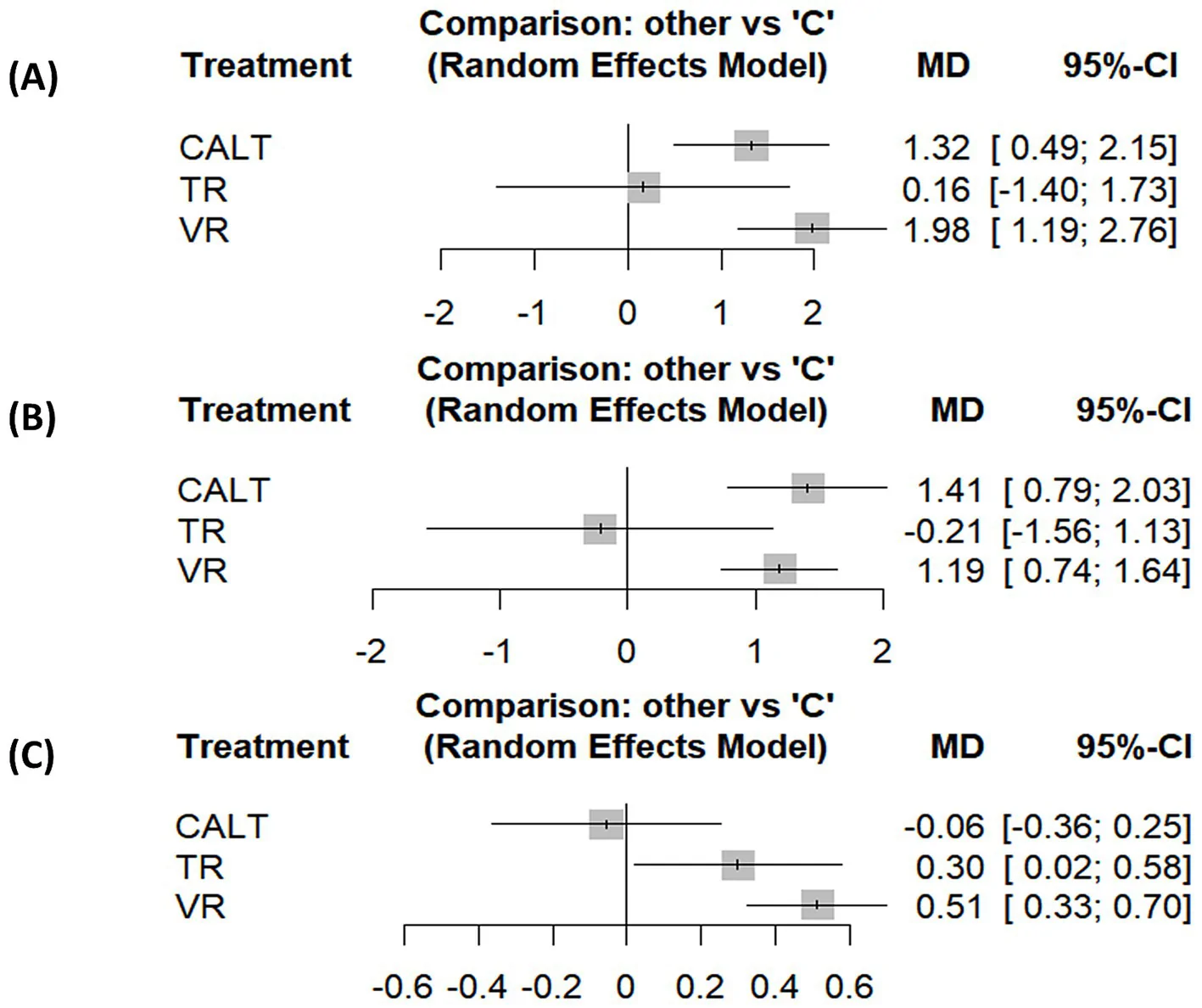
Forest plot for repetition **(A)**, naming **(B)**, and SAQOL-39 **(C)**. C, conventional therapy (speech and language therapy, routine rehabilitation training, and nursing care); CALT, computer-assisted language therapy; VR, virtual reality; TR, telerehabilitation; MD, mean difference.

#### Naming

For naming, compared with C, CALT showed the greatest improvement (MD = 1.41, 95% CI 0.79 to 2.03; SUCRA = 89.87%), followed by VR (MD = 1.19, 95% CI 0.74 to 1.64; SUCRA = 75.38%). No significant benefit was observed with TR. (Figure 5B; Table 6).

**Table 6:** League table for the naming (lower left) and SAQOL-39 (upper right) showing the mean differences in scores and their 95% CIs among the interventions.

Treatment	SAQOL-39			
Naming	C^a^	−0.30 (−0.58,−0.02)	−0.06 (−0.36, 0.25)	−0.51 (−0.70, −0.33)
	−0.21 (−1.56, 1.13)	TR^b^	−0.36 (−0.77, 0.06)	−0.21 (−0.55, 0.12)
	−1.41 (−2.03, −0.79)	−1.62 (−3.11,−0.14)	CALT^c^	−0.57 (−0.93, −0.21)
	−1.19 (−1.64,−0.74)	−1.40 (−2.83, −0.02)	−0.22 (−0.99, 0.55)	VR^d^

^a^C, conventional therapy (speech and language therapy, routine rehabilitation training, and nursing care); ^b^TR, telerehabilitation; ^c^CALT, computer-assisted language therapy; ^d^VR, virtual reality.

#### Stroke and aphasia quality of life Scale-39 (SAQOL-39)

In the SAQOL-39 assessment, VR showed the greatest improvement (MD = 0.51, 95% CI 0.33 to 0.70; SUCRA = 96.40%). TR ranked second (MD = 0.30, 95% CI 0.02 to 0.58; SUCRA = 68.08%). CALT, however, showed no significant benefit. (Figure 5C; Table 6).

#### Intervention rankings

The intervention rankings are as follows: aphasia quotient (AQ): VR > CALT>TR > C; spontaneous speech: VR > CALT>TR > C; auditory comprehension: VR > CALT>TR > C; repetition: VR > CALT>TR > C; naming: CALT>VR > C > TR; SAQOL−39: VR > TR > C > CALT. See Figure 6 for a stacked diagram of the included studies.

**Figure 6:**
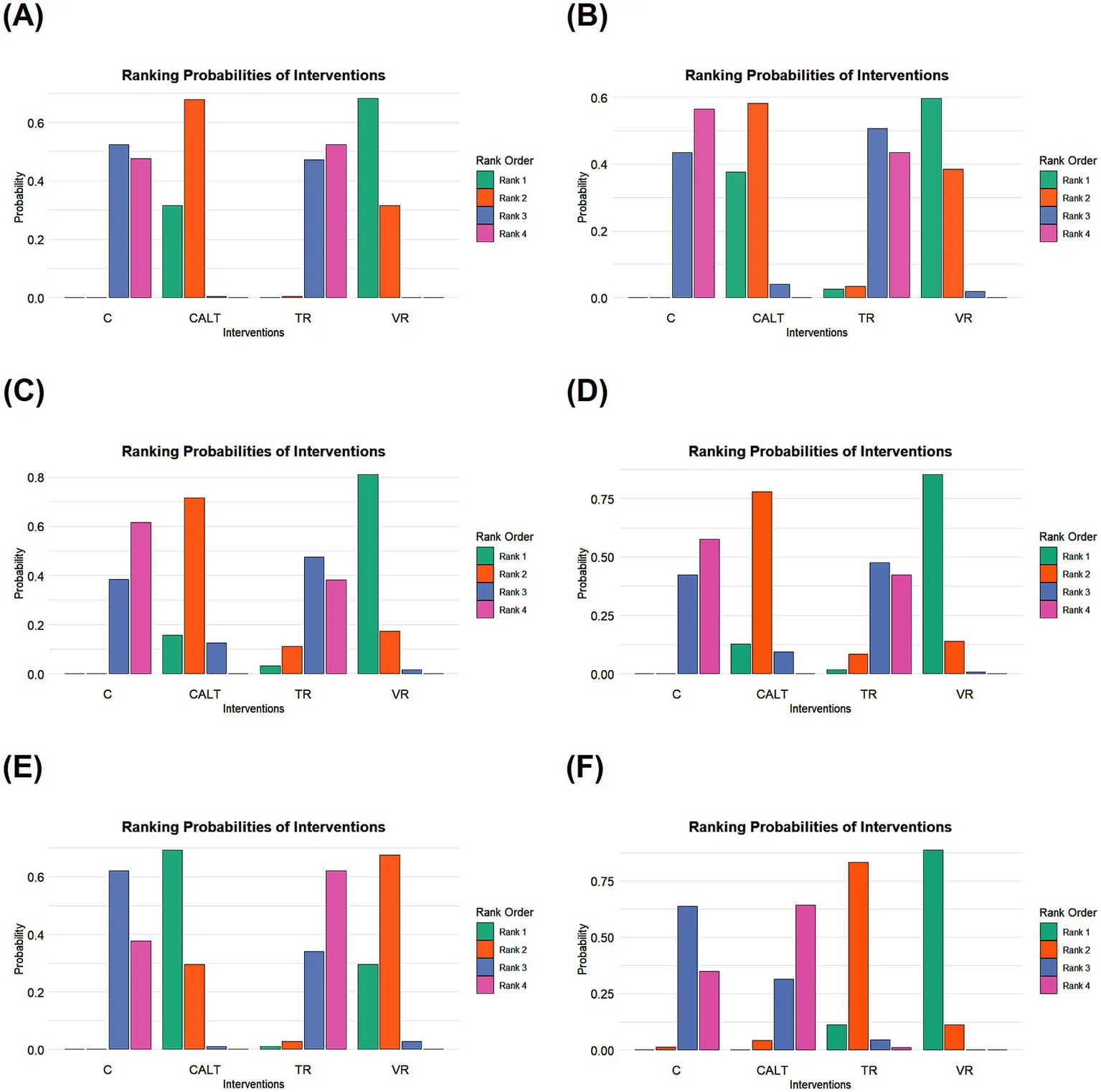
Stacked chart of included studies. C: conventional therapy (speech and language therapy, routine rehabilitation training, and nursing care); CALT, computer-assisted language therapy; VR, virtual reality; TR, telerehabilitation; MD, mean difference. **(A)** Aphasia quotient; **(B)** spontaneous speech; **(C)** auditory comprehension; **(D)** repetition; **(E)** naming; **(F)** SAQOL-39.

#### Key comparisons

According to the league table (Tables 4–6), based on aphasia quotient scores, VR was significantly superior to C (MD = −10.77, 95% CI − 12.06 to −9.48), and CALT was significantly superior to C (MD = −10.01, 95% CI − 12.77 to −7.25), while there was no significant difference between TR and C (MD = −0.31, 95% CI − 7.16 to 6.54), and CALT was significantly superior to TR (MD = −10.32, 95% CI − 17.70 to −2.94), VR was significantly superior to TR (MD = −11.08, 95% CI − 18.05 to −4.11). Based on spontaneous speech scores, VR was significantly superior to C (MD = −2.35, 95% CI − 2.99 to −1.71), CALT was significantly superior to C (MD = −2.22, 95% CI − 3.02 to −1.42), and there was no significant difference between TR and C (MD = −0.18, 95% CI − 2.47 to 2.11). CALT may be superior to TR (MD = −2.04, 95% CI − 4.46 to −0.19), and VR may be superior to TR (MD = −2.17, 95% CI − 4.55 to −0.20). Based on auditory comprehension scores, VR was significantly superior to C (MD = −1.47, 95% CI − 2.13 to −0.81), and CALT was significantly superior to C (MD = −1.01, 95% CI − 1.69 to −0.34), while there was no significant difference between TR and C (MD = −0.19, 95% CI − 1.50 to 1.12). VR may be superior to TR (MD = −1.28, 95% CI − 2.74 to −0.07); comparisons between other interventions (e.g., CALT vs. TR, CALT vs. VR) did not show any statistically significant differences. Based on the repetition score, VR was significantly superior to C (MD = −1.98, 95% CI − 2.76 to −1.19), CALT was significantly superior to C (MD = −1.32, 95% CI − 2.15 to −0.49), and there was no significant difference between TR and C (MD = −0.16, 95% CI − 1.73 to 1.40). VR may be superior to TR (MD = −1.81, 95% CI − 3.57 to −0.06); comparisons between other interventions (e.g., CALT vs. VR, CALT vs. TR) showed no statistically significant differences. Based on naming scores, CALT was significantly superior to C (MD = −1.41, 95% CI − 2.03 to −0.79), and VR was significantly superior to C (MD = −1.19, 95% CI − 1.64 to −0.74); there was no significant difference between TR and C (MD = −0.21, 95% CI − 1.56 to 1.13), CALT may have been superior to VR (MD = −1.62, 95% CI − 3.11 to −0.14), VR may be superior to TR (MD = −1.40, 95% CI − 2.83 to −0.02), and no statistically significant difference was observed between CALT and VR. Based on SAQOL−39 scores, VR was significantly superior to C (MD = −0.51, 95% CI − 0.70 to −0.33); TR may be superior to C (MD = −0.30, 95% CI − 0.58 to −0.02), while there was no significant difference between CALT and C (MD = −0.06, 95% CI − 0.36 to 0.25). Comparisons between other interventions (e.g., CALT vs. TR, VR vs. TR) showed no statistically significant differences. With the exception of the SAQOL-39 score, differences between TR and C were not statistically significant (CI contains 0).

### Heterogeneity analysis

Heterogeneity across the included studies was assessed using the I^2^ statistic. For the aphasia quotient, spontaneous speech, and SAQOL-39, the I^2^ values were 2.7, 25.3, and 33.5% respectively, indicating low heterogeneity. In contrast, the auditory comprehension, repetition, and naming domains exhibited greater heterogeneity (I^2^ = 87.1, 80.4, and 57.4%, respectively), indicating high between-study heterogeneity (Supplementary File 5).

The potential causes of this phenomenon are discussed in depth below from a clinical perspective.

#### Heterogeneity of intervention programs

Differences in intervention dosage and duration: Intervention duration across the included studies ranged from 2 weeks to 6 months, with varying weekly intervention frequencies. Different language functions may have different levels of sensitivity to intervention duration, and this discrepancy may lead to variations in intervention effects.

Differences in the integration of intervention techniques: In VR Marshall 2016, a custom immersive VR social communication training system based on real−life situations was adopted, while in Xu (43), a VR system based on mirror neuron action observation therapy was employed.

#### Differences in baseline population characteristics

Regarding the severity of aphasia, the patients included in different studies exhibited substantial variations in baseline language impairment, ranging from mild to severe aphasia. Differences in patients’ linguistic repertoires, educational levels, and bilingual backgrounds may all be potential sources of heterogeneity.

With regard to stroke type and lesion location, damage to different brain regions (e.g., the inferior frontal gyrus, superior temporal gyrus, or subcortical conduction tracts) may lead to different aphasia subtypes (e.g., motor, sensory, or conduction aphasia). Patients with different subtypes may show significant differences in both their neuroplastic responses to specific language rehabilitation strategies (e.g., auditory comprehension training and expressive language training) and the degree of benefit they gain from these approaches. This may account for the substantial variations in intervention outcomes observed across studies. Given the high level of heterogeneity detected in the analysis of certain language functions, clinical interventions should fully account for a patient’s aphasia type, intervention duration, lesion location, and stage of disease progression. Clinicians need to strike a comprehensive balance between efficacy evidence and clinical feasibility to develop personalized rehabilitation plans. Further research is required to investigate the sources of heterogeneity through sensitivity analyses, subgroup analyses, and subgroup regression analyses.

### Subgroup analysis and meta-regression

To understand which aspects of digital interventions may affect the functional improvement outcomes of patients with PSA, and which factors contribute to heterogeneity among included studies, we conducted a subgroup analysis on outcomes with high heterogeneity. See Supplementary File 6. Specifically, subgroup analyses were stratified by disease and intervention duration, and meta-regressions were performed for disease duration, intervention time, and patient age. However, quantitative analyses for baseline aphasia severity (e.g., mild vs. severe), specific syndromes (e.g., Broca’s, Wernicke’s), or distinct technological protocols could not be performed. Most primary studies lacked the necessary stratified or individual participant data, reporting only aggregated outcomes for mixed populations.

#### Disease duration

Subgroup analyses stratified by disease duration demonstrated that the treatment group consistently outperformed the control group in auditory comprehension, repetition, and naming tasks. Notably, patients in the≤3-month subgroup generally achieved superior outcomes compared to those in the>3-month subgroup, suggesting that digital interventions may confer greater clinical benefits when initiated early after stroke. This finding aligns with the established concept that neuroplasticity is more robust and the potential for language reorganization is heightened during the early post-stroke period. However, significant heterogeneity persisted within the≤3-month subgroup, indicating that stratification by disease duration alone was insufficient to fully resolve between-study heterogeneity. Therefore, while disease duration is a crucial contributing factor, it does not independently account for all the observed heterogeneity. Beyond disease duration, other variables—including aphasia subtype, lesion location, intervention timing, and variations in intervention protocols—may also contribute to the heterogeneity in treatment outcomes. The next step should be to further explore the sources of heterogeneity using methods such as sensitivity analysis and subgroup regression analysis.

#### Intervention time

For auditory comprehension, the intervention group outperformed the control group overall (MD = 1.12, 95% CI 0.66 to 1.59); however, this effect varied substantially by intervention duration. Short-term interventions (2, 4, or 8 weeks) were more effective than medium- and long-term interventions (3 or 6 months), with the 4-week intervention yielding the largest effect (MD = 1.78, 95% CI 1.10 to 2.46). This finding suggests that longer intervention duration does not necessarily translate into greater efficacy. For the repetition outcome, intervention duration appeared to modulate efficacy: 4-week and 3-month interventions produced the largest effects, followed by 6-month and 8-week interventions, whereas 2-week and 10-week interventions did not confer a clear advantage. However, despite the pronounced effect of the 4-week intervention, the overall effect estimate remains uncertain due to high heterogeneity. For naming, the 3-month intervention demonstrated the greatest efficacy (MD = 2.13, 95% CI 1.59 to 2.67), followed by the 6-month and 8-week interventions; short-term interventions (2 or 4 weeks) showed comparatively weaker effects. Overall, naming improvements increased progressively with longer intervention duration.

All three language domains exhibited time-dependent effects. Overall, the greatest improvements were observed when interventions lasted between 8 weeks and 3 months, whereas the shorter 2-week intervention yielded more limited benefits. Although some benefits were seen with certain 6-month interventions, these results should be interpreted cautiously, given the limited number of included studies. Statistically significant differences were observed across all subgroups, indicating that intervention duration may be an important effect modifier. This finding underscores the importance of sustained, sufficient intervention intensity for promoting language recovery in PSA.

#### Meta-regression

Meta-regression indicated that disease duration, age, and intervention duration did not significantly moderate the efficacy of digital interventions on language outcomes in PSA. However, for naming outcomes, the efficacy of VR increased significantly with longer intervention duration. Nevertheless, given the small number of long-term VR studies and the potential for confounding by heterogeneity in VR protocols, training intensity, and patients’ baseline impairment, this finding should be interpreted cautiously. Therefore, large-scale, standardized, long-term studies are needed to further validate the observed dose–response relationship for VR intervention duration (Supplementary File 7).

### Sensitivity analysis

To assess the robustness of our results, we conducted a stepwise exclusion sensitivity analysis using R 4.5.2 software, in which we sequentially excluded each of the included RCTs. The results showed that the impact of different studies on the overall heterogeneity varied significantly across different language function measures. For auditory comprehension, excluding the study by Xu (43) reduced heterogeneity from 87 to 68.76%, suggesting that this study was the primary source of heterogeneity. However, even after excluding this study, heterogeneity remained at a relatively high level, indicating that between-study variation for this measure was not caused by a single study, but rather by the combined effects of multiple factors, including intervention protocols, baseline impairment levels, and the mechanisms of digital interventions. Therefore, the overall results of this study demonstrate good robustness. For repetition, after excluding the Xu (43) study, we found that heterogeneity decreased from 80 to 52.1%, suggesting that this study was the primary source of heterogeneity for this outcome. The high heterogeneity may be attributed to the fact that the Xu (43) study included only patients with Broca’s aphasia. Broca’s area overlaps significantly with the human mirror neuron system, and the VR-based speech-action observation training employed in that study activates Broca’s area to improve patients’ language function, which differs from the intervention mechanisms of other included studies. For naming, after excluding the study by Li (29), we found that heterogeneity decreased from 57 to 35.1%, suggesting that this study was the primary source of heterogeneity for this outcome. The high level of heterogeneity may be attributed to the fact that Li (29) employed a multimodal intervention combining immersive VR with transcranial direct current stimulation (tDCS); the extent to which this synergistic intervention promotes language function remodeling far exceeds that of other single-modal digital interventions (Supplementary File 8).

### Publication bias

The funnel plots for each index showed a symmetrical distribution, indicating no significant publication bias. The Egger test results further confirmed this conclusion (aphasia quotient *p* = 0.3589; spontaneous speech *p* = 0.1769; auditory comprehension *p* = 0.1205; repetition *p* = 0.0489; naming *p* = 0.7085).

It should be noted that for SAQOL-39, the limited number of included original studies led to insufficient statistical power for the Egger test, preventing reliable results from being obtained. Therefore, this study only qualitatively assessed the publication bias of this index based on the symmetry of the funnel plot. The funnel plot of this scale showed that the effect points of the included studies were approximately symmetrically distributed on both sides of the combined effect size, and no obvious bias was detected. Accordingly, this index was considered to have a low risk of publication bias (see Figure 7).

**Figure 7:**
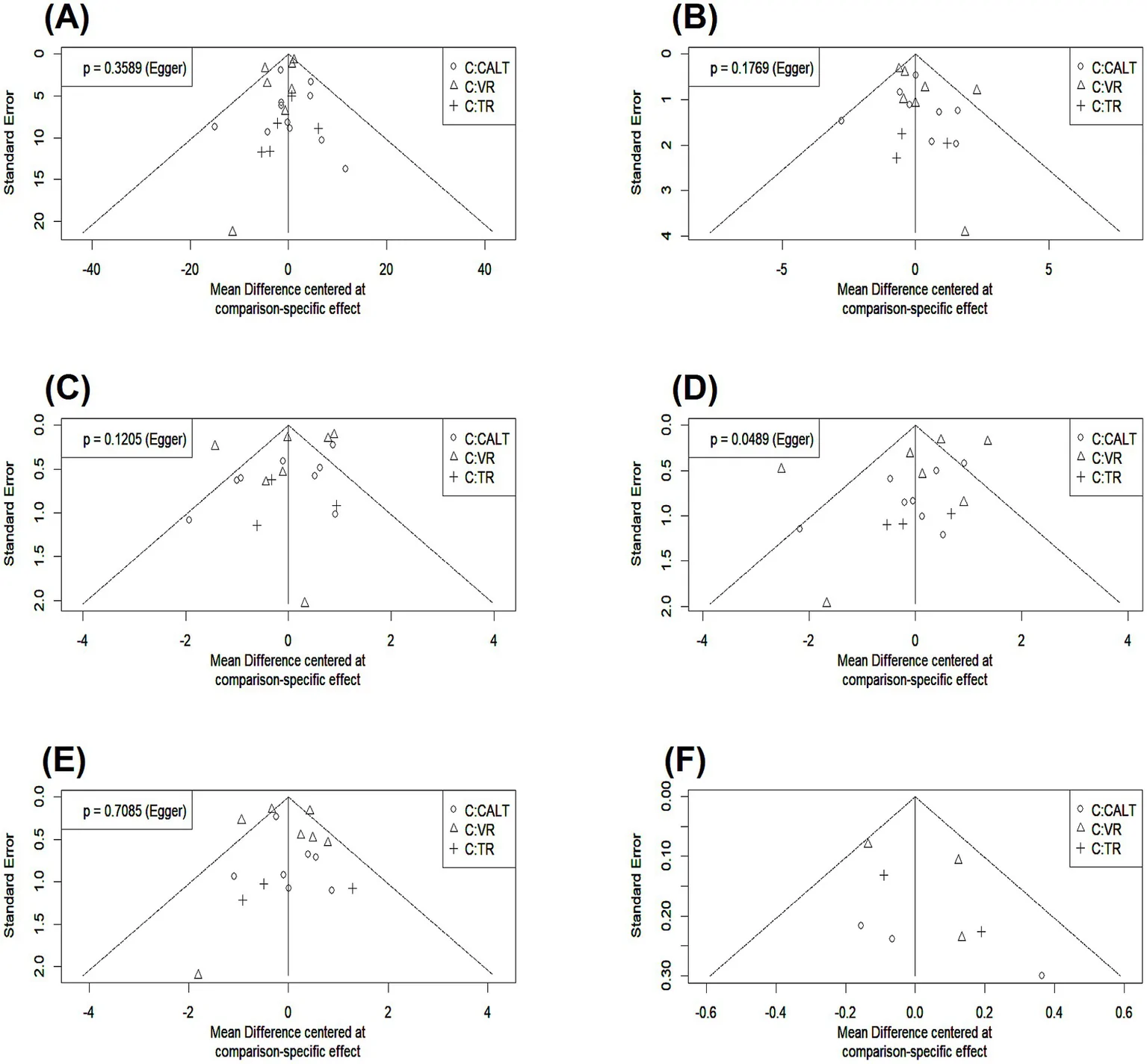
Funnel chart of the inclusion literature. C, conventional therapy (speech and language therapy, routine rehabilitation training, and nursing care); CALT, computer-assisted language therapy; VR, virtual reality; TR, telerehabilitation; MD, mean difference. **(A)** Aphasia quotient; **(B)** spontaneous speech; **(C)** auditory comprehension; **(D)** repetition; **(E)** naming; **(F)** SAQOL−39.

## Discussion

### Summary

Post-stroke aphasia is a complex acquired language dysfunction that occurs after stroke (47). It usually manifests as impairment of language comprehension and expressive ability (48), which seriously weakens patients’ self-identity, impairs their daily communication and social participation, and ultimately leads to restricted activities of daily living (5). The emergence of digital intervention technologies including CALT, VR, and TR has opened up a new direction for PSA assessment and precision intervention, owing to their advantages of multimodal stimulation, high-intensity repetitive training, and the ability to break space–time limits. However, different digital intervention technologies have distinct characteristics in their mechanisms of promoting cerebral neuroplasticity and functional reorganization of the language network. Clinically, when faced with patients’ complex speech impairments (such as difficulties in spontaneous speech, repetition, or naming), there is still a lack of systematic integration of the advantages and disadvantages of different digital intervention schemes across multidimensional language outcomes. To the best of our knowledge, this study is the first network meta-analysis to simultaneously evaluate and comprehensively compare the efficacy of three common digital interventions—computer-assisted intervention, VR intervention, and telerehabilitation for PSA. A total of 25 studies involving 1,069 patients were included. This study not only covers core language dimension indicators including Aphasia Quotient (AQ), spontaneous speech, auditory comprehension, repetition, and naming, but also incorporates the SAQOL-39. It aims to evaluate the effects of different digital therapies on different language function impairments and reduced quality of life, and to rank them appropriately according to efficacy. This will not only help reduce the blindness of clinical selection of digital intervention schemes, but also provide an evidence-based basis for personalized precision rehabilitation of patients with PSA.

### Principal findings

The results show that for the above three digital therapies, different digital interventions do not have absolute advantages, but instead demonstrate obvious functional specificity. VR showed the best clinical efficacy in AQ, spontaneous speech, auditory comprehension, repetition, and SAQOL-39, while CALT performed the best in improving naming function. Although TR does not show significant advantages in core language function, it has certain value in improving patients’ quality of life. These results indicate that digital intervention is not simply a substitute for traditional language therapy, but acts on different language domains through distinct mechanisms. Therefore, the selection of digital interventions in clinical practice should fully consider the characteristics of patients’ language impairments.

AQ is the core measure for assessing aphasia severity and overall language function on the WAB, encompassing multiple dimensions such as spontaneous speech, auditory comprehension, repetition, and naming. For improving AQ, VR ranked highest in SUCRA, indicating that it is most likely to benefit overall language recovery, possibly by promoting language network reorganization. Although a meta-analysis by Cao et al. (18) confirmed that VR improves AQ in patients with PSA, it found no significant effect on other language functions. This finding was consistent with our NMA, with both analyses showing a beneficial effect of VR on AQ. However, the magnitude of the effect estimates cannot be directly compared because of differences in the included studies, comparator groups, and analytical approaches. Cao et al. (18) compared VR with conventional therapy, whereas our NMA evaluated the relative effects of multiple digital interventions within a network framework. The lack of significant effects on other language functions in Cao et al. (18) may have been influenced by the small number of included studies (*n* = 7), limited sample sizes, and clinical heterogeneity among the included populations. Previous studies have shown that recovery from PSA depends not only on the restitution of local brain function but also, to a large extent, on the functional reorganization of language networks (49, 50). VR can integrate high-intensity, multisensory stimulation (e.g., visual, auditory, and motor feedback) into language training, creating an enriched communication environment. Such an enriched environment has been shown to promote experience-dependent neuroplasticity after stroke (51), thereby facilitating the reconstruction of damaged neural function and ultimately improving overall language function in patients with PSA.

To improve spontaneous speech, auditory comprehension, repetition, and SAQOL-39 scores, VR ranked first in SUCRA, highlighting its unique advantages in promoting functional communication and quality of life. A meta-analysis by Zhang et al. (52) showed that VR improved spontaneous speech, auditory comprehension, repetition, and naming in patients with PSA, which was consistent with our findings for spontaneous speech, auditory comprehension, and repetition. For naming, however, the findings differed: Zhang et al. (52) reported a significant benefit of VR, whereas CALT ranked first in our NMA. These differences may reflect variations in the available evidence and the relative effects of the interventions. The aforementioned functions reflect different aspects of language abilities: expression, comprehension, and repetition. However, they all depend on intact language input, processing, and output processes, and they share a common neural substrate. Therefore, in this study, we combine discussions of the potential mechanisms through which VR improves these language functions. From a behavioral perspective, traditional aphasia rehabilitation often relies on decontextualized, repetitive training. Due to the lack of authentic pragmatic context, treatment gains achieved in the clinic often fail to transfer to daily communication. In contrast, VR can create communication environments that closely resemble real-life situations and can be tailored to patients’ individual needs and abilities (53). VR provides strong immersion, is highly customizable and easy to use, which enhances patients’ motivation for language training (54). It facilitates a shift from passive training to active participation and increases training engagement; higher engagement typically yields greater language improvements. A systematic review (55) showed that the immersive communication environment provided by VR increases opportunities for language use, enhances communication initiative and training participation, and thereby significantly improves communication skills. Giachero et al. (15) compared VR-based daily living conversation therapy with conventional conversation therapy and found that only the VR group showed significant improvements in auditory comprehension and repetition. Marshall et al. (14) used the EVA Park platform to create highly immersive virtual spaces (e.g., supermarkets, restaurants) and embedded language training into daily activities such as shopping or socializing. This approach enhanced the ecological validity of language therapy and significantly improved functional communication, which may explain the observed gains in spontaneous speech and auditory comprehension. Second, patients with PSA often experience severe communication anxiety and social isolation. VR can offer a safe, stress-free interactive space where patients can experience positive support and a sense of security during multi-person interactions. Amaya et al. (56) showed that within the virtual environment of EVA Park, participants typically reported greater pleasure, engagement, and relaxation, which helped reduce social isolation and enhanced communication confidence, thereby improving language function and quality of life. These studies once again proved the reliability of the results of this network meta-analysis.

From a neurophysiological perspective, current research on VR in PSA rehabilitation has predominantly focused on behavioral language outcomes, whereas the underlying neural mechanisms remain relatively unexplored within the included studies. Therefore, existing mechanistic explanations for VR’s effects cannot be directly derived from our network meta-analysis and largely rely on indirect inferences from neuroplasticity principles and functional imaging studies. According to Wernicke’s hypothesis (57), two principal language pathways exist in the left dominant hemisphere (58), a widely accepted dual-pathway model supported by evidence from multiple studies (59–61). The dorsal language pathway is involved in phonological processing and speech production, supporting auditory-motor mapping (59, 62), and is crucial for repetition and articulation (58, 63). Whereas the ventral language pathway is primarily responsible for semantic processing (sound-to-meaning mapping) (59, 62), playing an essential role in language comprehension and semantically driven speech output (57, 62). Different aphasia types reflect pathway-specific impairments: dorsal pathway damage often leads to conduction aphasia (characterized by repetition deficits) or motor aphasia (laborious, nonfluent spontaneous speech), whereas ventral pathway damage typically results in sensory aphasia (impaired auditory comprehension) (62, 64), findings consistent with the dual-pathway model. Based on this theoretical framework, we theoretically speculate that VR may create an enriched training environment by integrating multimodal stimuli including visual, auditory, and motor feedback. Such enriched stimuli may activate the impaired ventral pathway, thereby improving auditory comprehension. Concurrently, the high-frequency interaction and real-time feedback in VR may enhance sensorimotor integration within the dorsal pathway, potentially facilitating the recovery of spontaneous speech and repetition. In addition, VR may improve language function in patients with PSA by enhancing compensatory right-hemisphere activation. Several neuroimaging studies have shown that contralateral homologues of left-hemisphere language areas are activated after stroke (65–67), and some researchers believe that the enhanced activation and altered functional connectivity in the homologous brain region and surrounding areas of the right hemisphere are associated with better language performance (68, 69). A recent neuroimaging study also reported compensatory activation of right-hemisphere language homologues in patients with left-hemisphere PSA (70). Notably, the right hemisphere has inherent advantages in processing visuospatial information, emotional expression, and face recognition (71). VR can construct realistic, three-dimensional life scenes or game environments. Rich spatial and emotional cues may enhance right-hemisphere activity and promote activation of its language homologues, thereby facilitating compensatory language improvement in patients.

VR may also improve language function in patients with PSA by activating the mirror neuron system (MNS). Several studies have shown that the core network of mirror neurons overlaps significantly with Broca’s area, which is responsible for speech production and expression in the brain (72, 73). Broca’s area is activated when patients observe or attempt to imitate specific actions, thereby improving language function. Through a randomized controlled trial combining fMRI, Chen et al. (74) found that, when observing and mimicking dynamic hand movements or vocal mouth patterns, the MNS core areas, such as the left inferior frontal gyrus (Broca’s area), were significantly activated in patients with aphasia. The patients’ self-speech and naming functions significantly improved compared to observing static pictures. However, because VR can provide multisensory stimuli with a higher level of immersion and a sense of stereo compared to traditional static picture recognition and simple gesture observation, we hypothesized that it may be more effective in activating core MNS areas, such as Broca’s area, which may in turn improve speech and other language functions in patients with PSA. Nevertheless, it is important to note that many of these neurophysiological and anatomical mechanisms are largely based on inferences from the external literature and should therefore be interpreted with caution.

In terms of improving naming scores, CALT ranked first in SUCRA, suggesting that it may be potentially the most effective digital therapy for enhancing naming ability, possibly because it targets activation of the residual language network in the left hemisphere. A randomized controlled trial by Braley et al. (28) showed that PSA patients who received digital therapy interventions demonstrated significant improvements in naming and word-finding abilities on standardized language tests, which is consistent with the findings of this network meta-analysis. The primary reasons why CALT improves naming function in PSA patients are as follows: First, CALT relies on multimedia technology to stimulate the brain through high-intensity, repetitive, and targeted semantic and speech training tasks, which can activate core language areas surrounding lesions in the left hemisphere (e.g., the inferior frontal gyrus), thereby enhancing patients’ naming ability (75). A functional magnetic resonance imaging (fMRI) study by Fridriksson et al. (76) found that improvements in patients’ naming abilities following targeted computer-assisted naming therapy were associated with activation of the residual language network in the left hemisphere (primarily in the left frontal and parietal regions). In a large-scale, multicenter randomized controlled trial, Palmer et al. (16) found that patients receiving computer-assisted speech therapy showed significant improvements in word recognition compared to the standard care group. These large-scale clinical studies further support the reliability of the findings in this meta-analysis. Furthermore, through multimedia applications such as computers or tablets, the difficulty of intervention tasks can be adaptively and individually tailored, and real-time feedback can be provided (77). A network meta-analysis noted (78) that a high-frequency, high-intensity, and sustained cumulative intervention dose is a key factor in achieving functional communication recovery, such as naming ability, in aphasia. Kurland et al. (79) also demonstrated that high-intensity repetitive practice is a prerequisite for inducing experience-dependent neuroplasticity in the brain, and tablet-based computer interventions provide the required high-dose therapy (80), thereby significantly improving patients’ naming abilities. Furthermore, by accurately identifying patients’ lexical retrieval deficits and quantitatively recording their training data, CALT overcomes the limitations of traditional outpatient settings in terms of treatment duration and geographic location, enabling longer and more intensive interventions that help sustain patients’ long-term rehabilitation benefits (81).

In this study, TR did not demonstrate superior efficacy across any of the core language measures in the WAB, and several scores showed no significant difference compared to conventional intervention. These results suggest that the primary role of TR may lie in overcoming physical spatial limitations rather than promoting functional reorganization of the brain’s language networks. Cacciante et al. (20) showed no significant differences in outcomes between TR and traditional face-to-face speech and language therapy in areas such as the AQ, auditory comprehension, and naming, which is consistent with the findings of this study. Possible reasons for the lack of significant clinical efficacy of TR include the fact that most current TR intervention programs primarily use communication or information technology to provide language stimuli equivalent to those of traditional speech-language therapy, and there is no apparent difference between the two in terms of the extent of language impairment recovery. Furthermore, remote interventions limit therapists’ ability to utilize nonverbal cues, and older stroke patients often experience cognitive decline and difficulties operating devices, which to some extent affect the quality of intervention and patients’ adherence to training. At the methodological level, the small sample sizes in the TR intervention trials included in this study and the wide confidence intervals for effect sizes may also have led to insufficient statistical power.

Although TR has no significant effect on treating language impairment, it ranks second in the SAQOL-39 score and performs significantly better than conventional interventions. This result objectively reflects that the core clinical value of TR lies more in improving patients’ communication self-efficacy and psychosocial adaptation level, rather than directly repairing damaged language pathways. Home-based training overcomes space and traffic barriers and reduces the economic burden on caregivers. Furthermore, it shortens hospital stays, promotes the extension of professional rehabilitation services to communities, and significantly improves the accessibility of rehabilitation resources. For discharged patients in the chronic phase, clinical efficacy evaluation should not be limited to traditional indicators of language function. TR has effectively improved patients’ quality of life through continuous rehabilitation guidance, broken space constraints, reduced family medical expenses, and optimized the allocation of medical resources. In future clinical practice, TR can be used as a basic platform combined with other digital therapies according to patients’ personalized needs to improve both patients’ language function and quality of life.

### Clinical implications

To bridge the gap between our statistical findings and clinical practice, and acknowledging the limitations of current evidence, we propose a preliminary personalized, multidimensional clinical decision-making framework (Figure 8).

**Figure 8:**
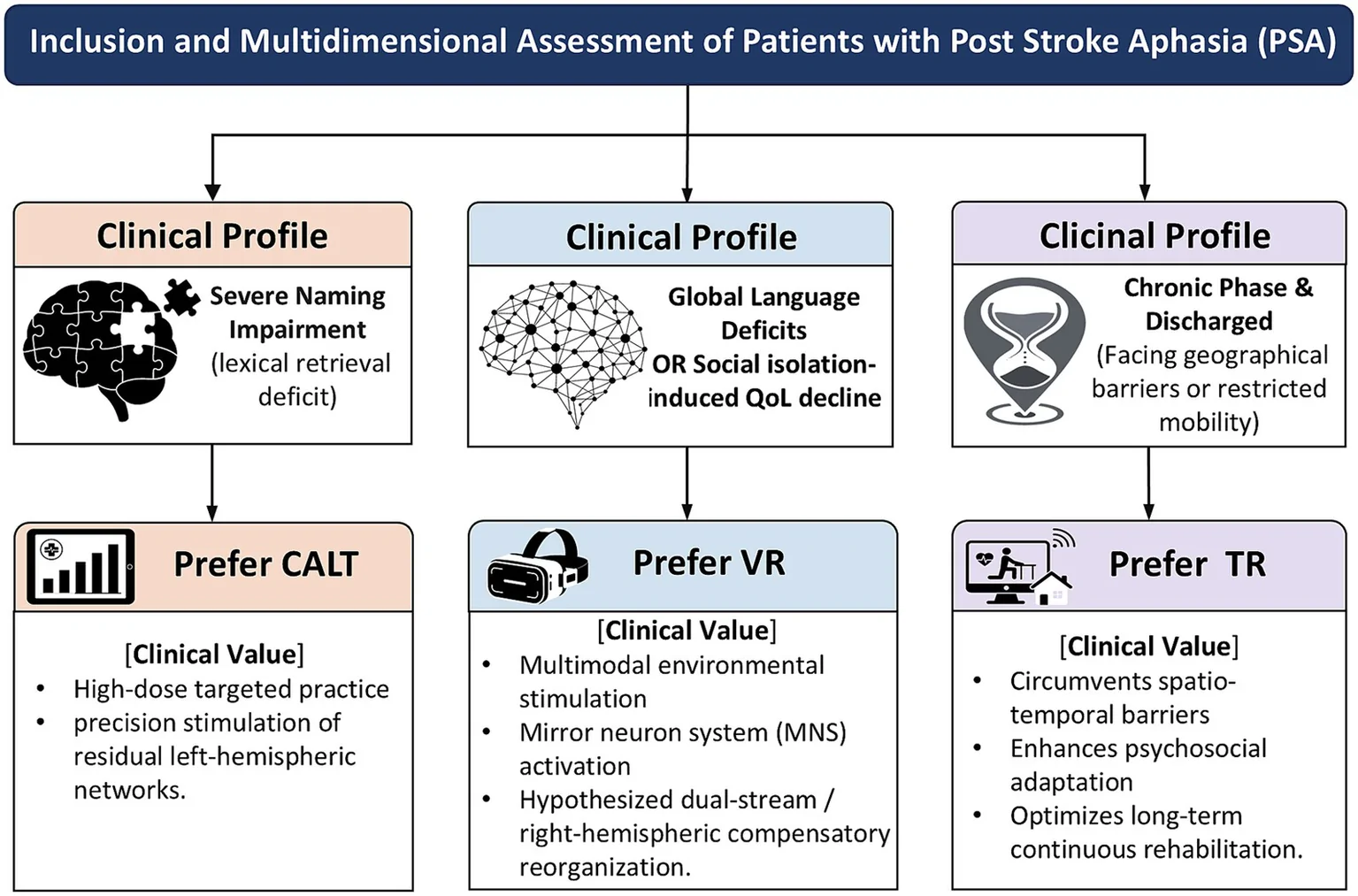
Clinical decision-making framework for selecting digital interventions for patients with post-stroke aphasia (PSA). The flowchart illustrates the decision-making framework for matching digital interventions with the clinical characteristics of PSA patients: (left): For patients with severe naming impairments; (middle): For patients with global language deficits (including AQ reduction, spontaneous speech impairment, auditory comprehension impairment, and repetition impairment) OR decreased quality of life; (right): It is aimed at patients in the chronic phase who face geographical location or mobility constraints. The bottom panel details the specific clinical value and therapeutic mechanism of each modality and points out the potential dual-pathway model activation of VR and the potential compensatory reorganization mechanism of the right hemisphere. CALT, computer-assisted language therapy, VR: virtual reality, TR: telerehabilitation, QoL: quality of life. (The figure is created by author YS).

This framework emphasizes that the selection of digital interventions requires a high degree of personalization to match patients’ specific clinical characteristics with optimal treatment options. Specifically, CALT may be considered for patients who primarily present with naming dysfunction, such as severe vocabulary retrieval impairment. VR appears to be a viable candidate for patients who show deficits in overall language function (AQ, spontaneous speech, auditory comprehension, and repetition disorders) or experience a decline in quality of life due to social isolation. TR represents a practical alternative for discharged patients in the chronic phase who reside in remote areas or have limited mobility. Given the low certainty of evidence, these suggestions should be interpreted cautiously as preliminary references rather than definitive clinical guidelines. This framework aims to optimize the allocation of clinical resources and assist in the delivery of rehabilitation benefits for patients with PSA.

### Limitations

This study provides valuable insights into the efficacy of digital therapies for post-stroke aphasia (PSA), but several limitations should be acknowledged. First, the GRADE framework rated the quality of evidence as low across all comparisons, primarily due to the following interrelated issues: (1) Risk of bias: approximately 36% of the included studies exhibited a high risk of bias, particularly in blinding of participants/personnel and allocation concealment. For example, unblinded therapists may have inadvertently influenced patient engagement in VR or CALT interventions, potentially inflating effect estimates for these therapies compared with C. (2) Imprecision: wide confidence intervals in key comparisons reflect limited statistical power, likely attributable to the sparse network structure and the substantial contribution of indirect evidence to the pooled estimates. Additionally, the number of TR studies was limited (*n* = 5), and only three of these studies reported complete data for the individual WAB subdomains, which may have contributed to further imprecision in the corresponding analyses. Similarly, the small number of studies evaluating SAQOL-39 precluded a reliable statistical assessment of publication bias using Egger’s test. Although the funnel plot showed an approximately symmetrical distribution, the possibility of publication bias remains. (3) Inconsistency: Heterogeneity persisted despite sensitivity analyses, likely attributable to variations in intervention protocols and population characteristics.

Specifically, for VR-based interventions, differences in technological approaches (e.g., immersive, semi-immersive, and mirror neuron-based systems) may have contributed to heterogeneity in treatment effects. Potential conceptual overlap among CALT, VR, and TR may also have contributed to clinical heterogeneity, despite classification according to the primary active components of each intervention. Because TR often functions primarily as a delivery method rather than a standalone intervention, it may overlap conceptually with other digital interventions. Despite these criteria, residual overlap may still introduce subtle clinical heterogeneity and obscure the distinct effects of each intervention type. Therefore, comparative effect estimates and network rankings should be interpreted with caution. Furthermore, the intervention durations varied considerably across studies (ranging from 2 weeks to 6 months), which may have limited the ability to assess potential dose–response relationships and complicated comparisons across interventions.

Clinically, differences in population characteristics (e.g., inclusion of both subacute and chronic stroke phases, and aphasia types ranging from motor to global aphasia) may have further contributed to heterogeneity. Because the primary studies lacked sufficient data to perform subgroup analyses by aphasia syndrome and baseline severity, our pooled estimates represent an average treatment effect across clinically heterogeneous populations. This may obscure the potential influence of these clinical differences on treatment outcomes; for example, the demands of VR-based interventions may differ significantly between patients with severe global aphasia and those with milder fluent aphasia. Furthermore, incomplete reporting of some study characteristics, often labeled as “not otherwise reported” (NOR), may have limited the assessment of potential sources of heterogeneity and the interpretation of the findings.

The generalizability of the findings may also be limited by the geographic distribution of the available evidence. Most studies were conducted in China and English-speaking countries, which may restrict the applicability of the results to other healthcare systems and cultural contexts. Although no language restrictions were applied, variations in rehabilitation practices and cultural backgrounds should be considered when interpreting the findings.

These limitations have important implications for clinical implementation. Although CALT and VR demonstrated domain-specific advantages in naming and other language outcomes, respectively, the low GRADE scores suggest that these findings should be interpreted with caution. For example, the apparent superiority of VR in spontaneous speech, auditory comprehension, and repetition may be influenced by its integration of multisensory stimulation and gamified feedback, which may engage social brain networks, enhance patient motivation, and synergistically improve language function—a mechanism that has not been isolated in the current VR trials.

Finally, most included studies evaluated outcomes only immediately after the intervention, with a lack of long-term follow-up data. Therefore, the sustainability of treatment effects and the long-term effectiveness of digital therapies for PSA remain uncertain.

### Further direction

Given these limitations, future research should focus on the following areas. First, it is crucial to conduct long-term follow-up studies. This will help to evaluate the sustained impact of digital therapies on patients with PSA and assess their long-term value in delaying language decline, improving quality of life, and promoting social reintegration. Second, future studies should further optimize intervention protocols, including clarifying the optimal intervention dose, frequency, and duration for different aphasia subtypes and language domains, as well as establishing the clinical basis for individualized treatment plans. In addition, combining neuromodulation techniques (e.g., transcranial magnetic stimulation) or traditional rehabilitation approaches (e.g., acupuncture, acupoint pressing) with digital therapies for synergistic application may yield more significant rehabilitation benefits, which is worth exploring in depth. Finally, from the perspective of technological development, with the continuous advancement of AI, VR, and other technologies, how to ensure that digital therapies reduce costs and maintain therapeutic efficacy through continuous technological updating and optimization is also an important area of research. For example, in resource-limited or geographically remote settings, CALT delivered via tablet or computer devices can provide a cost-effective and accessible language training program. And reinforcement learning algorithms can be integrated into VR-based language training modules to dynamically adjust task difficulty based on real-time performance. Future research should develop smarter adaptive training systems, more immersive VR scenarios, and more accurate tools for monitoring and analyzing language data in patients, in order to further improve the effectiveness and feasibility of digital therapies in the field of PSA rehabilitation.

## Conclusion

Overall, digital therapies provide preliminary evidence supporting personalized solutions for PSA rehabilitation. Among these, VR demonstrates domain-specific advantages in AQ, spontaneous speech, auditory comprehension, repetition, and quality of life, whereas CALT shows superiority in naming. These findings align with the study’s aims to quantify relative efficacy and identify the potentially optimal strategies for specific language domains. However, heterogeneity and methodological limitations in existing trials necessitate further validation through high-quality RCTs with standardized protocols, larger samples, and long-term follow-ups. Clinical translation requires balancing efficacy, safety, and cost-effectiveness to advance precision rehabilitation strategies for PSA.

## Data Availability

The original contributions presented in the study are included in the article/Supplementary material, further inquiries can be directed to the corresponding author.
