# Supplementary material for: Efficacy of digital interventions for patients with post-stroke aphasia: a systematic review and network meta-analysis
Source: Front Neurol. 2026 Oct 12;17:1936067. doi: 10.3389/fneur.2026.1936067 (PMC13696256; doi:10.3389/fneur.2026.1936067)
Supplement: Supplementary file 1 [file Table_1.docx]

**Search Strategy**

**Pubmed**

| **#** | **Search terms** | **Results** |
| --- | --- | --- |
| 1 | Stroke[MeSH Terms] | 200,253 |
| 2 | stroke[Title/Abstract] OR Strokes[Title/Abstract] OR Cerebrovascular Accident[Title/Abstract] OR Cerebrovascular Accidents[Title/Abstract] OR Cerebral Stroke[Title/Abstract] OR Cerebral Strokes[Title/Abstract] OR Stroke, Cerebral[Title/Abstract] OR Strokes, Cerebral[Title/Abstract] OR Cerebrovascular Apoplexy[Title/Abstract] OR Apoplexy, Cerebrovascular[Title/Abstract] OR Vascular Accident, Brain[Title/Abstract] OR Brain Vascular Accident[Title/Abstract] OR Brain Vascular Accidents[Title/Abstract] OR Vascular Accidents, Brain[Title/Abstract] OR Cerebrovascular Stroke[Title/Abstract] OR Cerebrovascular Strokes[Title/Abstract] OR Stroke, Cerebrovascular[Title/Abstract] OR Strokes, Cerebrovascular[Title/Abstract] OR Apoplexy[Title/Abstract] OR Acute Strokes[Title/Abstract] OR Strokes, Acute[Title/Abstract] OR Cerebrovascular Accident, Acute[Title/Abstract] OR Acute Cerebrovascular Accident[Title/Abstract] OR Acute Cerebrovascular Accidents[Title/Abstract] | 399,212 |
| 3 | Aphasia[MeSH Terms] | 14,225 |
| 4 | ((aphasia[Title/Abstract] OR (aphasia, Broca[Title/Abstract])) OR (aphasia, Wernicke[Title/Abstract]) OR aphasias[Title/Abstract] OR dysphasia[Title/Abstract] OR dysphasias[Title/Abstract] OR anomia[Title/Abstract] OR anomias[Title/Abstract] OR language impairment[Title/Abstract] OR language impairments[Title/Abstract] OR language disorder[Title/Abstract] OR language disorders[Title/Abstract] OR speech disorder[Title/Abstract] OR speech disorders[Title/Abstract]) | 32,892 |
| 5 | Therapy, Computer-Assisted[MeSH Terms] | 50,766 |
| 6 | computer-assisted therapy[Title/Abstract] OR computer-assisted therapies[Title/Abstract] OR computer-assisted training[Title/Abstract] OR computer-assisted rehabilitation[Title/Abstract] OR computer-based therapy[Title/Abstract] OR computer-based training[Title/Abstract] OR computerized training[Title/Abstract] OR computerized therapy[Title/Abstract] OR tablet-based therapy[Title/Abstract] OR tablet-based training[Title/Abstract] OR tablet-based rehabilitation[Title/Abstract] OR ipad-based therapy[Title/Abstract] OR ipad-based training[Title/Abstract] OR smartphone application[Title/Abstract] OR smartphone applications[Title/Abstract] OR smartphone app[Title/Abstract] OR smartphone apps[Title/Abstract] OR mobile application[Title/Abstract] OR mobile applications[Title/Abstract] OR mobile app[Title/Abstract] OR mobile apps[Title/Abstract] OR digital therapeutic[Title/Abstract] OR digital therapeutics[Title/Abstract] OR dtx[Title/Abstract] OR software[Title/Abstract] OR computer program[Title/Abstract] OR computer programs[Title/Abstract] OR web-based therapy[Title/Abstract] OR web-based training[Title/Abstract] OR internet-based therapy[Title/Abstract] OR online platform[Title/Abstract] OR online platforms[Title/Abstract] | 349,798 |
| 7 | Virtual Reality[MeSH Terms] | 10,436 |
| 8 | Reality, Virtual[Title/Abstract] OR Virtual Reality, Educational[Title/Abstract] OR Educational Virtual Realities[Title/Abstract] OR Educational Virtual Reality[Title/Abstract] OR Reality, Educational Virtual[Title/Abstract] OR Virtual Realities, Educational[Title/Abstract] OR Virtual Reality, Instructional[Title/Abstract] OR Instructional Virtual Realities[Title/Abstract] OR Instructional Virtual Reality[Title/Abstract] OR Realities, Instructional Virtual[Title/Abstract] OR Reality, Instructional Virtual[Title/Abstract] OR Virtual Realities, Instructional[Title/Abstract] OR Virtual Reality[Title/Abstract] | 27,579 |
| 9 | Telemedicine[MeSH Terms] | 57,420 |
| 10 | telemedicine[Title/Abstract] OR telemedicines[Title/Abstract] OR telerehabilitation[Title/Abstract] OR tele-rehabilitation[Title/Abstract] OR telehealth[Title/Abstract] OR tele-health[Title/Abstract] OR teletherapy[Title/Abstract] OR teletherapies[Title/Abstract] OR tele-therapy[Title/Abstract] OR tele-therapies[Title/Abstract] OR remote consultation[Title/Abstract] OR remote consultations[Title/Abstract] OR remote rehabilitation[Title/Abstract] OR remote rehabilitations[Title/Abstract] OR online rehabilitation[Title/Abstract] OR online rehabilitations[Title/Abstract] OR online therapy[Title/Abstract] OR online therapies[Title/Abstract] OR web-based rehabilitation[Title/Abstract] OR web-based rehabilitations[Title/Abstract] OR internet-based rehabilitation[Title/Abstract] OR internet-based rehabilitations[Title/Abstract] OR video consultation[Title/Abstract] OR video consultations[Title/Abstract] OR video conference[Title/Abstract] OR video conferencing[Title/Abstract] OR mobile health[Title/Abstract] OR mhealth[Title/Abstract] OR m-health[Title/Abstract] OR ehealth[Title/Abstract] OR e-health[Title/Abstract] OR home-based telerehabilitation[Title/Abstract] OR home-based tele-rehabilitation[Title/Abstract] | 86,214 |
| 11 | #1 OR #2 | 438,552 |
| 12 | #3 OR #4 | 36,746 |
| 13 | #5 OR #6 | 397,247 |
| 14 | #7 OR #8 | 29,189 |
| 15 | #9 OR #10 | 106,285 |
| 16 | #11 AND #12 | 7,495 |
| 17 | #13 OR #14 OR #15 | 515,948 |
| 18 | #16 AND #17 | 253 |

**Cochrane Library**

| **#** | **Search terms** | **Results** |
| --- | --- | --- |
| 1 | MeSH descriptor: [Stroke] explode all trees | 19,474 |
| 2 | Stroke OR strokes OR Cerebrovascular Accident OR Cerebrovascular Accidents OR Cerebral Stroke OR Cerebral Strokes OR Stroke, Cerebral OR Strokes, Cerebral OR Cerebrovascular Apoplexy OR Apoplexy, Cerebrovascular OR Vascular Accident, Brain OR Brain Vascular Accident OR Brain Vascular Accidents OR Vascular Accidents, Brain OR Cerebrovascular Stroke OR Cerebrovascular Strokes OR Stroke, Cerebrovascular OR Strokes, Cerebrovascular OR Apoplexy OR Acute Strokes OR Strokes, Acute OR Cerebrovascular Accident, Acute OR Acute Cerebrovascular Accident OR Acute Cerebrovascular Accidents | 100,618 |
| 3 | #1 OR #2 | 101,011 |
| 4 | MeSH descriptor: [Aphasia] explode all trees | 779 |
| 5 | aphasia OR aphasia, Broca OR aphasia, Wernicke OR aphasias OR dysphasia OR dysphasias OR anomia OR anomias OR language impairment OR language impairments OR language disorder OR language disorders OR speech disorder OR speech disorders | 296,672 |
| 6 | #4 OR #5 | 296,672 |
| 7 | MeSH descriptor: [Therapy, Computer-Assisted] explode all trees | 3,322 |
| 8 | computer-assisted therapy OR computer-assisted therapies OR computer-assisted training OR computer-assisted rehabilitation OR computer-based therapy OR computer-based training OR computerized training OR computerized therapy OR tablet-based therapy OR tablet-based training OR tablet-based rehabilitation OR ipad-based therapy OR ipad-based training OR smartphone application OR smartphone applications OR smartphone app OR smartphone app OR mobile application OR mobile applications OR mobile app OR mobile apps OR digital therapeutic OR digital therapeutics OR dtx OR software OR computer program OR computer programs OR web-based therapy OR web-based training OR internet-based therapy OR online platform OR online platforms | 121,760 |
| 9 | #7 OR #8 | 122,584 |
| 10 | MeSH descriptor: [Virtual Reality] explode all trees | 1,829 |
| 11 | Reality, Virtual OR Virtual Reality, Educational OR Educational Virtual Realities OR Educational Virtual Reality OR Reality, Educational Virtual OR Virtual Realities, Educational OR Virtual Reality, Instructional OR Instructional Virtual Realities OR Instructional Virtual Reality OR Realities, Instructional Virtual OR Reality, Instructional Virtual OR Virtual Realities, Instructional OR Virtual Reality | 10,505 |
| 12 | #10 OR #11 | 10,525 |
| 13 | MeSH descriptor: [Telemedicine] explode all trees | 6,218 |
| 14 | Telemedicine OR telemedicines OR telerehabilitation OR tele-rehabilitation OR telehealth OR tele-health OR teletherapy OR teletherapies OR tele-therapy OR tele-therapies OR remote consultation OR remote consultations OR remote rehabilitation OR remote rehabilitations OR online rehabilitation OR online rehabilitations OR online therapy OR online therapies OR web-based rehabilitation OR web-based rehabilitations OR internet-based rehabilitation OR internet-based rehabilitations OR video consultationOR video consultations OR video conference OR video conferencing OR mobile health OR mhealth OR m-health OR ehealth OR e-health OR home-based telerehabilitation OR home-based tele-rehabilitation | 78,524 |
| 15 | #13 OR #14 | 78,582 |
| 16 | #3 AND #6 | 17,362 |
| 17 | #9 OR #12 OR #15 | 174,437 |
| 18 | #16 AND #17 | 2,072 |

**Web of Science**

| **#** | **Search terms** | **Results** |
| --- | --- | --- |
| 1 | TS=(Strokes OR Cerebrovascular Accident OR Cerebrovascular Accidents OR Cerebral Stroke OR Cerebral Strokes OR Stroke, Cerebral OR Strokes, Cerebral OR Cerebrovascular Apoplexy OR Apoplexy, Cerebrovascular OR Vascular Accident, Brain OR Brain Vascular Accident OR Brain Vascular Accidents OR Vascular Accidents, Brain OR Cerebrovascular Stroke OR Cerebrovascular Strokes OR Stroke, Cerebrovascular OR Stroke) | 455,546 |
| 2 | TS=(aphasia OR aphasia, Broca OR aphasia, Wernicke OR aphasias OR dysphasia OR dysphasias OR anomia OR anomias OR language impairment OR language impairments OR language disorder OR language disorders OR speech disorder OR speech disorders OR communicative disorder OR communicative disorders) | 83,632 |
| 3 | TS=(computer-assisted therapy OR computer-assisted therapies OR computer-assisted training OR computer-assisted rehabilitation OR computer-based therapy OR computer-based training OR computerized training OR computerized therapy OR tablet-based therapy OR tablet-based training OR tablet-based rehabilitation OR ipad-based therapy OR ipad-based training OR smartphone application OR smartphone applications OR smartphone app OR smartphone app OR mobile application OR mobile applications OR mobile app OR mobile apps OR digital therapeutic OR digital therapeutics OR dtx OR software OR computer program OR computer programs OR web-based therapy OR web-based training OR internet-based therapy OR online platform OR online platforms) | 806,934 |
| 4 | TS=(Reality, Virtual OR Virtual Reality, Educational OR Educational Virtual Realities OR Educational Virtual Reality OR Reality, Educational Virtual OR Virtual Realities, Educational OR Virtual Reality, Instructional OR Instructional Virtual Realities OR Instructional Virtual Reality OR Realities, Instructional Virtual OR Reality, Instructional Virtual OR Virtual Realities, Instructional OR Virtual Reality) | 51,466 |
| 5 | TS=(Telemedicine OR telemedicines OR telerehabilitation OR tele-rehabilitation OR telehealth OR tele-health OR teletherapy OR teletherapies OR tele-therapy OR tele-therapies OR remote consultation OR remote consultations OR remote rehabilitation OR remote rehabilitations OR online rehabilitation OR online rehabilitations OR online therapy OR online therapies OR web-based rehabilitation OR web-based rehabilitations OR internet-based rehabilitation OR internet-based rehabilitations OR video consultationOR video consultations OR video conference OR video conferencing OR mobile health OR mhealth OR m-health OR ehealth OR e-health OR home-based telerehabilitation OR home-based ) | 159,808 |
| 6 | #1 AND #2 | 8,624 |
| 7 | #3 OR #4 OR #5 | 979,501 |
| 8 | #6 AND #7 | 655 |

**Scopus(N=90)**

TITLE-ABS-KEY ( ( stroke OR strokes OR cerebrovascular accident OR cerebrovascular accidents OR cerebral stroke OR cerebral strokes ) AND ( aphasia OR aphasias OR dysphasia OR dysphasias OR anomia OR anomias OR language impairment OR language disorder) AND ( computer-assisted therapy OR computer-assisted training OR mobile application OR mobile applications OR mobile app OR mobile apps OR digital therapeutic OR digital therapeutics OR dtx OR virtual reality OR VR OR telemedicine OR telerehabilitation OR telehealth OR teletherapy OR artificial intelligence OR AI OR video game OR video games OR serious game OR serious games OR gamification ) )

EMbase

| **#** | **Search terms** | **Results** |
| --- | --- | --- |
| 1 | 'cerebrovascular accident'/exp OR 'cerebrovascular accident' | 558,518 |
| 2 | (stroke:ti,ab OR strokes:ti,ab OR 'cerebrovascular accident':ti,ab OR 'cerebrovascular accidents':ti,ab OR 'cerebral stroke':ti,ab OR 'cerebral strokes':ti,ab OR 'cerebral infarction':ti,ab OR 'cerebral infarctions':ti,ab OR 'brain infarction':ti,ab OR 'brain infarctions':ti,ab OR apoplexy:ti,ab) | 659,897 |
| 3 | #1 OR #2 | 795,557 |
| 4 | 'aphasia'/exp OR 'anomia'/exp | 44,477 |
| 5 | (aphasia:ti,ab OR aphasias:ti,ab OR 'aphasia broca':ti,ab OR 'aphasia wernicke':ti,ab OR dysphasia:ti,ab OR dysphasias:ti,ab OR anomia:ti,ab OR anomias:ti,ab OR 'language impairment':ti,ab OR 'language impairments':ti,ab OR 'language disorder':ti,ab OR 'language disorders':ti,ab OR 'speech disorder':ti,ab OR 'speech disorders':ti,ab) | 47,185 |
| 6 | #4 OR #5 | 64,002 |
| 7 | 'computer assisted therapy'/exp OR 'computer assisted therapy' | 15,539 |
| 8 | ('computer assisted therapy':ti,ab OR 'computer assisted therapies':ti,ab OR 'computer assisted training':ti,ab OR 'computerized training':ti,ab OR 'computerized therapy':ti,ab OR 'tablet based therapy':ti,ab OR 'tablet based training':ti,ab OR 'ipad based therapy':ti,ab OR 'mobile application':ti,ab OR 'mobile applications':ti,ab OR 'mobile app':ti,ab OR 'mobile apps':ti,ab OR 'smartphone application':ti,ab OR 'smartphone applications':ti,ab OR 'digital therapeutic':ti,ab OR 'digital therapeutics':ti,ab OR dtx:ti,ab OR software:ti,ab OR 'web based training':ti,ab OR 'internet based therapy':ti,ab OR 'online platform':ti,ab) | 545,957 |
| 9 | #7 OR #8 | 560,454 |
| 10 | 'virtual reality'/exp OR 'virtual reality' | 52,395 |
| 11 | ('reality virtual':ab,ti OR 'virtual reality educational':ab,ti OR 'educational virtual realities':ab,ti OR 'educational virtual reality':ab,ti OR 'reality educational virtual':ab,ti OR 'virtual realities educational':ab,ti OR 'virtual reality instructional':ab,ti OR 'instructional virtual realities':ab,ti OR 'instructional virtual reality':ab,ti OR 'realities instructional virtual':ab,ti OR 'reality instructional virtual':ab,ti OR 'virtual realities instructional':ab,ti OR 'virtual reality':ab,ti) | 33,890 |
| 12 | #10 OR #11 | 52,413 |
| 13 | 'telemedicine'/exp OR 'telemedicine' | 112,199 |
| 14 | ('telemedicine':ab,ti OR 'telemedicines':ab,ti OR 'telerehabilitation':ab,ti OR 'tele-rehabilitation':ab,ti OR 'telehealth':ab,ti OR 'tele-health':ab,ti OR 'teletherapy':ab,ti OR 'teletherapies':ab,ti OR 'tele-therapy':ab,ti OR 'tele-therapies':ab,ti OR 'remote consultation':ab,ti OR 'remote consultations':ab,ti OR 'remote rehabilitation':ab,ti OR 'remote rehabilitations':ab,ti OR 'online rehabilitation':ab,ti OR 'online rehabilitations':ab,ti OR 'online therapy':ab,ti OR 'online therapies':ab,ti OR 'web-based rehabilitation':ab,ti OR 'web-based rehabilitations':ab,ti OR 'internet-based rehabilitation':ab,ti OR 'internet-based rehabilitations':ab,ti OR 'video consultation':ab,ti OR 'video consultations':ab,ti OR 'video conference':ab,ti OR 'video conferencing':ab,ti OR 'mobile health':ab,ti OR 'mhealth':ab,ti OR 'm-health':ab,ti OR 'ehealth':ab,ti OR 'e-health':ab,ti OR 'home-based telerehabilitation':ab,ti OR 'home-based':ab,ti) | 123,251 |
| 15 | #13 OR #14 | 179,529 |
| 16 | #3 AND #6 | 16,624 |
| 17 | #9 OR #12 OR #15 | 771,540 |
| 18 | #16 AND #17 | 616 |
| 19 | AND ('controlled study'/de OR 'randomized controlled trial'/de) | 250 |

**CNKI(N=475) (Chinese version)**

**((SU=脑卒中 + 脑卒中患者 + 脑卒中康复 + 脑血管意外 + 脑梗死 + 脑出血 + 缺血性脑卒中 + 缺血性脑卒中患者 +中风) AND (SU=失语 + 失语症 + 语言障碍 + 言语障碍 + 沟通障碍)) AND ((SU=计算机辅助 + 计算机辅助治疗 + 移动应用 + APP + 软件 + 平板电脑 ) OR (SU=虚拟现实 + 虚拟现实技术 + 虚拟现实系统 + '虚拟现实(vr)' + 虚拟现实技术应用 + '虚拟现实(vr)技术' + VR ) OR (SU= 远程康复 + 远程治疗 + 移动医疗 + 人工智能 + AI + 游戏 + 视频会议))**

**CNKI(N=475) (English version)**

**((SU=Stroke + stroke patients + stroke rehabilitation + cerebrovascular accident + cerebral infarction + cerebral hemorrhage + ischemic stroke + ischemic stroke patients + cerebrovascular stroke) AND (SU=aphasia + aphasia + language disorder + speech disorder + communication disorder)) AND ((SU=computer-assisted + computer-assisted therapy + mobile application + APP + software + tablet computer) OR (SU=virtual reality + virtual reality technology + virtual reality system + ‘virtual reality (VR)’ + application of virtual reality technology + “virtual reality (VR) technology” + VR) OR (SU=telerehabilitation + teletherapy + telemedicine + artificial intelligence + AI + games + videoconferencing))**
