# Supplementary material for: Efficacy of digital interventions for patients with post-stroke aphasia: a systematic review and network meta-analysis
Source: Front Neurol. 2026 Oct 12;17:1936067. doi: 10.3389/fneur.2026.1936067 (PMC13696256; doi:10.3389/fneur.2026.1936067)
Supplement: Supplementary file 2 [file Table_2.docx]

| Serial Number | Included Study | Unique Identifier | Supplementary Search Method | Specific Source of Retrieval | Source Article/Databases Identifier (PMID/DOI) |
| --- | --- | --- | --- | --- | --- |
| 1 | Marshall et al (1) 2020 | doi: 10.1371/journal.pone.0239715 | Citation searching | Retrieved from Reference List of Cao (2) et al 2021 | Matched in PubMed (PMID: 33834386) |
| 2 | zhou et al (3) 2018 | doi: 10.3389/fpsyg.2018.02312 | Citation searching | Retrieved from Reference List of Cacciante et al (4) 2021 | Matched in PubMed (PMID: 34052617) |
| 3 | Meltzer et al (5) 2017 | doi: 10.1080/02687038.2017.1355440 | Citation searching | Retrieved from Reference List of Cacciante et al (4) 2021 | Matched in PubMed (PMID: 34052617) |
| 4 | Braley et al (6) 2021 | doi: 10.3389/fneur.2021.626780 | Citation searching | Retrieved from Reference List of Jiang et al (7) 2024 | Matched in PubMed (PMID: 38933323) |
| 5 | Cherney et al (8) 2010 | doi: 10.1310/tsr1706-423 | Citation searching | Retrieved from Reference List of Jiang et al (7) 2024 | Matched in PubMed (PMID: 38933323) |
| 6 | Kesav et al (9) 2017 | doi: 10.1016/j.jns.2017.07.010 | Citation searching | Retrieved from Reference List of Jiang et al (7) 2024 | Matched in PubMed (PMID: 38933323) |
| 7 | Katz et al (10) 1997 | doi: 10.1044/jslhr.4003.493 | Citation searching | Retrieved from Reference List of Jiang et al (7) 2024 | Matched in PubMed (PMID: 38933323) |
| 8 | Chen et al (11) 2021 | doi: 10.3233/nre-210125 | Citation searching | Retrieved from Reference List of Zhang et al (12) 2023 | Matched in CNKI (DOI:10.3761/j.issn.0254-1769.2023.03.006) |
| 9 | You et al (13) 2019 | doi: 10.1159/000503960 | Citation searching | Retrieved from Reference List of Zhang et al (12) 2023 | Matched in CNKI (DOI:10.3761/j.issn.0254-1769.2023.03.006) |
| 10 | Xu et al (14) 2020 | URL:https://kns.cnki.net/kcms2/article/abstract?v=gW15YWgIns3qzm1Ejz_9RPn0YOpkugxV_gfNq7YV37qJvGFpqbPBZi4P-eLSnNFY0clNb9eN-EbNMbF_0tB1SdvsHyWKpRP_D_buMTLUDBxywiJeU_HvXR7lJdqg4hH17fQmNyp9KgiEgqF6dg538Pdc-sYqrz2QoeHQxg7elgKcWZDQPc-k2SAGDtLFYLZJ&uniplatform=NZKPT&language=CHS | Citation searching | Retrieved from Reference List of Zhang et al (12) 2023 | Matched in CNKI (DOI:10.3761/j.issn.0254-1769.2023.03.006) |
| 11 | Tian et al (15) 2017 | doi: 10.3969/j.issn.1001-1242.2017.10.013 | Citation searching | Retrieved from Reference List of Zhang et al (12) 2023 | Matched in CNKI (DOI:10.3761/j.issn.0254-1769.2023.03.006) |
| 12 | Katz et al (16) 1992 | doi: 10.1080/02687039208248588 | Google Scholar | Retrieved from Google Scholar | Matched in Scopus  (DOI:10.1080/02687039208248588) |

**Audit trail and primary database cross-referencing of candidate records identified via supplementary other methods.**

1.	Marshall J, Devane N, Talbot R, Caute A, Cruice M, Hilari K, et al. A randomised trial of social support group intervention for people with aphasia: A Novel application of virtual reality. PLoS One. (2020) 15 (9): e0239715. doi: 10.1371/journal.pone.0239715

2.	Cao Y, Huang X, Zhang B, Kranz GS, Zhang D, Li X, et al. Effects of virtual reality in post-stroke aphasia: a systematic review and meta-analysis. Neurol Sci. (2021) 42 (12): 5249-59. doi: 10.1007/s10072-021-05202-5

3.	Zhou QM, Lu X, Zhang Y, Sun ZH, Li JN, Zhu ZD. Telerehabilitation Combined Speech-Language and Cognitive Training Effectively Promoted Recovery in Aphasia Patients. Front Psychol. (2018) 9. doi: 10.3389/fpsyg.2018.02312

4.	Cacciante L, Kiper P, Garzon M, Baldan F, Federico S, Turolla A, et al. Telerehabilitation for people with aphasia: A systematic review and meta-analysis. J Commun Disord. (2021) 92: 106111. doi: 10.1016/j.jcomdis.2021.106111

5.	Meltzer JA, Baird AJ, Steele RD, Harvey SJ. Computer-based treatment of poststroke language disorders: a non-inferiority study of telerehabilitation compared to in-person service delivery. APHASIOLOGY. (2018) 32 (3): 290-311. doi: 10.1080/02687038.2017.1355440

6.	Braley M, Pierce JS, Saxena S, De Oliveira E, Taraboanta L, Anantha V, et al. A Virtual, Randomized, Control Trial of a Digital Therapeutic for Speech, Language, and Cognitive Intervention in Post-stroke Persons With Aphasia. Front Neurol. (2021) 12: 626780. doi: 10.3389/fneur.2021.626780

7.	Jiang Z, He M, Zhang C, Chen X. The effect of mobile application-based technology on post-stroke aphasia: a systematic review. Front Neurol. (2024) 15: 1405209. doi: 10.3389/fneur.2024.1405209

8.	Cherney LR. Oral Reading for Language in Aphasia (ORLA): Evaluating the Efficacy of Computer-Delivered Therapy in Chronic Nonfluent Aphasia. Top Stroke Rehabil. (2010) 17 (6): 423-31. doi: 10.1310/tsr1706-423

9.	Kesav P, Vrinda SL, Sukumaran S, Sarma PS, Sylaja PN. Effectiveness of speech language therapy either alone or with add-on computer-based language therapy software (Malayalam version) for early post stroke aphasia: A feasibility study. J Neurol Sci. (2017) 380: 137-41. doi: 10.1016/j.jns.2017.07.010

10.	Katz RC, Wertz RT. The efficacy of computer-provided reading treatment for chronic aphasic adults. J Speech Lang Hear Res. (1997) 40 (3): 493-507. doi: 10.1044/jslhr.4003.493

11.	Chen Q, Shen W, Sun H, Shen D, Cai X, Ke J, et al. Effects of mirror therapy on motor aphasia after acute cerebral infarction: A randomized controlled trial. NeuroRehabilitation. (2021) 49 (1): 103-17. doi: 10.3233/nre-210125

12.	Yuanxing Z, Lei H, Qiansheng J, Yufan H, Cuiling J, Lu C. Effects of virtual reality-based language training for people with post-stroke aphasia: a meta-analysis. Chin J Nurse (2023) 58 (3): 296-303. doi: 10.3761/j.issn.0254-1769.2023.03.006

13.	You L, Wang Y, Chen W, Zhang S, Rao J, Liu L, et al. The Effectiveness of Action Observation Therapy Based on Mirror Neuron Theory in Chinese Patients with Apraxia of Speech after Stroke. Eur Neurol. (2019) 81 (5-6): 278-86. doi: 10.1159/000503960

14.	Xu L, Luo H, Chen S, Li J, Li Y, Fu J, et al. Effect observation of mirror neuron-based speech action observation in the treatment of poststroke Broca aphasia. J Pract Hosp Clin Med. (2020) 17 (06): 139-42. doi:

15.	Tian L, Zhu H, Liu L, Rao J, Meng A, Zhang J. Effects of Action Observation Therapy Based on Mirror Neuron Theory on Non-Fluent Aphasia After Stroke[In Chinese] Chin J Rehabil Med. (2017) 32 (10): 1152-4. doi: 10.3969/j.issn.1001-1242.2017.10.013

16.	Katz RC, Wertz RT. Computerized hierarchical reading treatment in aphasia. Aphasiology. (1992) 6 (2): 165-77. doi: 10.1080/02687039208248588
