# Supplementary material for: Efficacy of digital interventions for patients with post-stroke aphasia: a systematic review and network meta-analysis
Source: Front Neurol. 2026 Oct 12;17:1936067. doi: 10.3389/fneur.2026.1936067 (PMC13696256; doi:10.3389/fneur.2026.1936067)
Supplement: Supplementary file 3 [file Table_3.docx]

# **GRADE Summary of Findings**

| Outcome | Evidence base | Effect / finding | Certainty | Key reasons |
| --- | --- | --- | --- | --- |
| Aphasia quotient（AQ） | 23 reports  （n = 948） | MD = 10.77 , 95% CI (9.48 ,12.06),  I²=2.7% , P= 0.42  VR ranks first  (SUCRA=89.57% | Moderate | Risk of Bias: Inherent lack of blinding for participants and therapists in rehabilitation interventions. |
| Spontanesous speech | 18 reports  （n = 786） | MD = 2.35 , 95% CI (1.71 ,2.99),  I²=25.3% , P= 0.16  VR ranks first  (SUCRA=85.45%) | Moderate | Risk of Bias: Insufficient reporting of allocation concealment and outcome assessor blinding. |
| Auditory comprehension | 18 reports  （n = 786） | MD = 1.47 , 95% CI (0.81 ,2.13),  I²=87.1% , P< 0.001  VR ranks first  (SUCRA=87.1%) | Low | Risk of Bias: Potential performance bias. Inconsistency: Very high statistical heterogeneity |
| Repetition | 18 reports  （n = 786） | MD = 1.98 , 95% CI (1.19 ,2.76),  I²=80.4% , P< 0.001  VR ranks first  (SUCRA=94.89%) | Low | Risk of Bias: Unclear randomization details. Inconsistency: Very high statistical heterogeneity |
| Naming | 17 reports  （n = 724） | MD = 1.41 , 95% CI (0.79 ,2.03),  I²=57.4% , P< 0.001  CALT ranks first  (SUCRA=89.87%) | Low | Inconsistency: Heterogeneity between software types. Imprecision: Reliance on indirect evidence for certain comparisons. |
| SAQOL-39 | 8 reports  （n = 315） | MD = 0.51 , 95% CI (0.33 ,0.70),  I²=33.5% , P= 0.18 VR ranks first (SUCRA=96.4%) | Low | Risk of Bias: Subjectivity of self-reported scales. Imprecision: Small number of studies and limited total sample size. |
