# Supplementary material for: Efficacy of digital interventions for patients with post-stroke aphasia: a systematic review and network meta-analysis
Source: Front Neurol. 2026 Oct 12;17:1936067. doi: 10.3389/fneur.2026.1936067 (PMC13696256; doi:10.3389/fneur.2026.1936067)
Supplement: Supplementary file 4 [file Table_4.docx]

WAB and SAQOL-39 Outcome Scores Reported in the Included Studies

| **Study (year) and group** | **Intervention** | **Sample size** | **AQ, mean (SD)** | **Spontaneous speech, mean (SD)** | **Auditory comprehension, mean (SD)** | **Repetition, mean (SD)** | **Naming, mean (SD)** | **SAQOL-39, mean (SD)** |
| --- | --- | --- | --- | --- | --- | --- | --- | --- |
| Kesav et al (23) (2017) |  |  |  |  |  |  |  |  |
| TG^a^ | CALT^b^ | 11 | 63.5 (33.8) | NOR^c^ | NOR | NOR | NOR | NOR |
| CG^d^ | C^e^ | 9 | 65.1 (27.6) | NOR | NOR | NOR | NOR | NOR |
| Alam et al (24) (2021) |  |  |  |  |  |  |  |  |
| TG | CALT | 5 | 62.76 (3.6) | 6.6 (0.89) | 9.67 (0.32) | 7.29 (0.7) | 7.81 (0.42) | 4.09 (0.24) |
| CG | C | 5 | 51.18 (2.37) | 4.4 (0.54) | 9.53 (0.39) | 5.49 (1.13) | 6.16 (0.3) | 3.99 (0.42) |
| Liu et al (25) (2022) |  |  |  |  |  |  |  |  |
| TG | CALT | 33 | 51.42 (23.46) | 9.31 (5.64) | 6.56 (2.47) | 6.37 (3.26) | 3.46 (3.12) | NOR |
| CG | C | 35 | 39.95 (23.9) | 7.97 (4.77) | 4.55 (2.7) | 5 (3.62) | 2.45 (2.5) | NOR |
| Cherney (26) (2010) |  |  |  |  |  |  |  |  |
| TG | CALT | 11 | 64.9 (18.7) | NOR | NOR | NOR | NOR | NOR |
| CG | C | 14 | 50.7 (27.6) | NOR | NOR | NOR | NOR | NOR |
| Gan et al (12) (2025) |  |  |  |  |  |  |  |  |
| TG | TR^f^ | 14 | 68.5 (11.64) | NOR | NOR | NOR | NOR | 3.47 (0.29) |
| CG | C | 14 | 69.41 (14.59) | NOR | NOR | NOR | NOR | 3.08 (0.4) |
| Marshall et al (27)(2020) |  |  |  |  |  |  |  |  |
| TG | VR^g^ | 16 | 81.86 (12.57) | NOR | NOR | NOR | NOR | 3.73 (0.72) |
|  |  |  |  |  |  |  |  |  |
| CG | C | 18 | 71.79 (12.27) | NOR | NOR | NOR | NOR | 3.35 (0.65) |
| Zhou et al (22) (2018) |  |  |  |  |  |  |  |  |
| **Study (year) and group** | **Intervention** | **Sample size** | **AQ, mean (SD)** | **Spontaneous speech, mean (SD)** | **Auditory comprehension, mean (SD)** | **Repetition, mean (SD)** | **Naming, mean (SD)** | **SAQOL-39, mean (SD)** |
| TG | CALT | 10 | 58.1 (19.9) | 10.6 (4.68) | 6.5 (1.6) | 7 (2.6) | 5.7 (2.3) | NOR |
| CG | C | 10 | 54.9 (25.7) | 9.9 (4.16) | 6.4 (2.80 | 6.2 (2.8) | 4.3 (2.5) | NOR |
| Zhou et al (22) (2018) |  |  |  |  |  |  |  |  |
| TG | TR | 10 | 55.3 (23.2) | 9.4 (4.59) | 6.5 (2.4) | 6.7 (2.0) | 5.1 (2.4) | NOR |
| CG | C | 10 | 50.1 (28.8) | 8.5 (5.54) | 5.7 (2.7) | 6.3 (2.80) | 4.4 (3.0) | NOR |
| Braley et al (28) (2021) |  |  |  |  |  |  |  |  |
| TG | TR | 17 | 68.37 (26.24) | 12.76 (5.78) | 8.36 (1.7) | 6.09 (2.9) | 6.97 (3.26) | 3.77 (0.56) |
| CG | C | 15 | 66.4 (20.22) | 12.07 (4.01) | 7.84 (1.8) | 6.6 (2.6) | 6.69 (2.51) | 3.66 (0.7) |
| Li et al (29) (2024) |  |  |  |  |  |  |  |  |
| TG | VR | 45 | 91.46 (8.65) | 18.46 (1.32) | 9.56 (0.36) | 8.84 (1.32) | 8.87 (1.03) | 3.73 (0.37) |
| CG | C | 45 | 75.94 (7.82) | 15.49 (1.75) | 8.98 (0.65) | 6.76 (1.64) | 6.74 (1.54) | 3.08 (0.39) |
| Chen et al (30) (2025) |  |  |  |  |  |  |  |  |
| TG | CALT | 11 | 64.44 (10.23) | 10.9 (2.46) | 8.27 (1.66) | 7.18 (1.2) | 7.09 (2.33) | 3.58 (0.64) |
| CG | C | 11 | 58.93 (13.06) | 10.27 (3.31) | 7.77 (0.96) | 6.25 (1.14) | 6.55 (2.81) | 3.57 (0.47) |
| Shao et al (31) (2025) |  |  |  |  |  |  |  |  |
| TG | VR | 53 | 75.92 (4.1) | 14.38 (2.35) | 7.56 (0.87) | 8.14 (0.95) | 7.88 (0.89) | NOR |
| CG | C | 53 | 66.23 (3.29) | 11.62 (1.7) | 6.86 (0.69) | 7.52 (0.89) | 7.12 (0.83) | NOR |
| Chen et al (32) (2025) |  |  |  |  |  |  |  |  |
| TG | CALT | 30 | NOR | 12.79 (3.18) | 7.93 (1.69) | 7.15 (1.72) | NOR | NOR |
| CG | C | 30 | NOR | 9.98 (3.26) | 6.81 (1.51) | 6.74 (1.52) | NOR | NOR |
| **Study (year) and group** | **Intervention** | **Sample size** | **AQ, mean (SD)** | **Spontaneous speech, mean (SD)** | **Auditory comprehension, mean (SD)** | **Repetition, mean (SD)** | **Naming, mean (SD)** | **SAQOL-39, mean (SD)** |
| Ye et al (33) (2021) |  |  |  |  |  |  |  |  |
| TG | VR | 30 | 69.86 (5.18) | 12.16 (3.11) | 7.51 (0.56) | 7.52 (0.68) | 7.52 (0.43) | NOR |
| CG | C | 30 | 59.82 (4.35) | 12.11 (3.08) | 6.02 (0.54) | 6.02 (0.58) | 6 (0.69) | NOR |
| Qian et al (34) (2023) |  |  |  |  |  |  |  |  |
| TG | CALT | 30 | 51.42 (23.46) | 9.34 (4.61) | 6.58 (2.13) | 6.53 (2.91) | 3.47 (3.08) | NOR |
| CG | C | 30 | 39.95 (23.9) | 6.91 (4.05) | 4.64 (2.51) | 5 (3.67) | 2.62 (2.39) | NOR |
| Xiong et al (35) (2021) |  |  |  |  |  |  |  |  |
| TG | CALT | 20 | 65.2 (26.74) | 12 (5.19) | 7.3 (4.27) | 8.5 (2.39) | 6.35 (2.57) | NOR |
| CG | C | 20 | 40.15 (28.27) | 7 (4.07) | 4.35 (2.32) | 5 (4.54) | 3.85 (3.3) | NOR |
| Katz et al (36) (1992) |  |  |  |  |  |  |  |  |
| TG | CALT | 15 | 75.01 (20.49) | NOR | NOR | NOR | NOR | NOR |
| CG | C | 15 | 65.35 (26.58) | NOR | NOR | NOR | NOR | NOR |
| Katz et al (37) (1997) |  |  |  |  |  |  |  |  |
| TG | CALT | 21 | 73.6 (22.6) | 13.8 (5.3) | 8.7 (1.2) | 7.3 (2.9) | 7 (2.4) | NOR |
| CG | C | 19 | 63.4 (28.5) | 12.2 (6.7) | 8.3 (1.8) | 6.1 (3.4) | 5.5 (3.3) | NOR |
| Peñaloza et al(38) (2021) |  |  |  |  |  |  |  |  |
| TG | TR | 8 | 73.6 (22.6) | NOR | NOR | NOR | NOR | NOR |
| CG | C | 8 | 63.4 (28.5) | NOR | NOR | NOR | NOR | NOR |
| Fuentes et al (39) (2024) |  |  |  |  |  |  |  |  |
| TG | CALT | 10 | 20.8 (8.9) | NOR | NOR | NOR | NOR | 3.5 (0.41) |
| CG | C | 11 | 15.4 (5.56) | NOR | NOR | NOR | NOR | 3.92 (0.9) |
| Kurul et al (40) (2026) |  |  |  |  |  |  |  |  |
| TG | VR | 46 | NOR | NOR | NOR | NOR | NOR | 3.16 (0.47) |
| **Stdy (year) and group** | **Intervention** | **Sample size** | **AQ, mean (SD)** | **Spontaneous speech, mean (SD)** | **Auditory comprehension, mean (SD)** | **Repetition, mean (SD)** | **Naming, mean (SD)** | **SAQOL-39, mean (SD)** |
| CG | C | 46 | NOR | NOR | NOR | NOR | NOR | 2.77 (0.55) |
| Chen et al (41) (2021) |  |  |  |  |  |  |  |  |
| TG | VR | 15 | 71 (41.74) | 11 (5.74) | 7.65 (3.06) | 7.95 (3.28) | 7.4 (3.56) | NOR |
| CG | C | 15 | 48.85 (71.31) | 10.5 (14.07) | 6.5 (7.28) | 4.3 (6.89) | 4.4 (7.32) | NOR |
| You et al (42) (2019) |  |  |  |  |  |  |  |  |
| TG | VR | 21 | NOR | 9.43 (3.17) | 7.12 (1.55) | 7 (1.54) | 5.21 (1.36) | NOR |
| CG | C | 21 | NOR | 6.62 (3.31) | 5.54 (1.93) | 5.15 (1.96) | 4.27 (1.57) | NOR |
| Xu et al (43) (2020) |  |  |  |  |  |  |  |  |
| TG | VR | 20 | 68.3 (14.44) | 12 (2.96) | 8.9 (1.02) | 8 (2.07) | 6.1 (0.96) | NOR |
| CG | C | 20 | 53.2 (6.52) | 10 (1.48) | 6 (0.37) | 3.5 (0.67) | 5.4 (1.93) | NOR |
| Tian et al (44) (2017) |  |  |  |  |  |  |  |  |
| TG | VR | 15 | 64.3 (19.03) | 9.1 (3.43) | 8.33 (1.78) | 8.45 (2.22) | 6.28 (1.42) | NOR |
| CG | C | 15 | 52.86 (18.52) | 6.75 (2.36) | 6.42 (1.77) | 7.38 (2.45) | 5.88 (1.54) | NOR |
| Meltzer et al (45) (2018) |  |  |  |  |  |  |  |  |
| TG | TR | 16 | 57.7 (24.9) | 11.6 (6.2) | 6.9 (2.35) | 5.8 (3) | 5.1 (2.8) | NOR |
| CG | C | 17 | 64.1 (23.5) | 12.6 (4.4) | 7.65 (2.65) | 5.1 (3) | 6.6 (3.1) | NOR |

^a^TG: treatment group, ^b^CALT: computer-assisted language therapy, ^c^NOR: not otherwise reported ^d^CG: control group, ^e^C: conventional therapy (speech and language therapy,
