# Supplementary material for: Efficacy of digital interventions for patients with post-stroke aphasia: a systematic review and network meta-analysis
Source: Front Neurol. 2026 Oct 12;17:1936067. doi: 10.3389/fneur.2026.1936067 (PMC13696256; doi:10.3389/fneur.2026.1936067)
Supplement: Supplementary file 5 [file Table_5.docx]

Heterogeneity Analysis(aphasia quotient)

Heterogeneity Analysis(spontaneous speech)

Heterogeneity Analysis(auditory comprehension)

Heterogeneity Analysis( repetition)

Heterogeneity Analysis( naming)

Heterogeneity Analysis( SAQOL-39)
