# Supplementary material for: Efficacy of digital interventions for patients with post-stroke aphasia: a systematic review and network meta-analysis
Source: Front Neurol. 2026 Oct 12;17:1936067. doi: 10.3389/fneur.2026.1936067 (PMC13696256; doi:10.3389/fneur.2026.1936067)
Supplement: Supplementary file 6 [file Table_6.docx]

Subgroup analysis(auditory comprehension)

Subgroup：Time

Subgroup：Duration

Subgroup analysis(repetition)

Subgroup：Time

Subgroup：Duration

Subgroup analysis(naming)

Subgroup：Time

Subgroup：Duration
