# Supplementary material for: Efficacy of digital interventions for patients with post-stroke aphasia: a systematic review and network meta-analysis
Source: Front Neurol. 2026 Oct 12;17:1936067. doi: 10.3389/fneur.2026.1936067 (PMC13696256; doi:10.3389/fneur.2026.1936067)
Supplement: Supplementary file 7 [file Table_7.docx]

Meta-regression（duration）

Auditory comprehension：CALT vs C

Auditory comprehension：VR vs C

Auditory comprehension：TR vs C

Repetition：CALT vs C

Repetition：VR vs C

Repetition：TR vs C

Naming：CALT vs C

Naming：VR vs C

Naming：TR vs C

Meta-regression（Age）

Auditory comprehension：CALT vs C

Auditory comprehension：VR vs C

Auditory comprehension：TR vs C

Repetition：CALT vs C

Repetition：VR vs C

Repetition：TR vs C

Naming：CALT VS C

Naming：VR VS C

Naming：TR VS C

Meta-regression（Time）

Auditory comprehension：CALT vs C

Auditory comprehension：VR vs C

Auditory comprehension：TR vs C

Repetition：CALT VS C

Repetition：VR vs C

Repetition：TR vs C

Naming： CALT vs C

Naming： VR vs C

Naming： TR vs C
